# Biological mechanisms of pulmonary inflammation and its association with seropositive rheumatoid arthritis

**DOI:** 10.3389/fimmu.2025.1530753

**Published:** 2025-05-23

**Authors:** Peiyue Yang, Yuqing Song, Mingwei Li

**Affiliations:** ^1^ Department of Rheumatism and Immunology, Fuxing Hospital affiliated to Capital Medical University, Beijing, China; ^2^ Department of Emergency, Suzhou Hospital of Traditional Chinese Medicine, Suzhou, China

**Keywords:** rheumatoid arthritis, pulmonary inflammation, anti-citrullinated protein antibodies, COVID-19, gut-lung axis

## Abstract

Although the pathogenesis of seropositive rheumatoid arthritis (RA) remains unclear, studies suggest that pulmonary inflammation-related biological mechanisms play a significant role in its development. This review thoroughly explores the mechanisms underlying early pulmonary lesions in seropositive RA, focusing on the mucosal barrier hypothesis, neutrophil extracellular traps, pathogenic microbial infections like COVID-19, Vitamin D, the microbiome and gut-lung axis, inhalation exposures and chronic pulmonary diseases. This study seeks to provide novel insights and theoretical foundations for the prevention and treatment of seropositive rheumatoid arthritis.

## 1 Introduction

Rheumatoid Arthritis (RA) is a systemic autoimmune disease manifesting as chronic inflammatory polyarthritis, which can ultimately lead to joint deformity and loss of workforce productivity (1). Abnormal protein citrullination and the formation of anti-citrullinated protein antibodies (ACPA) are critical pathogenic mechanisms in RA and are associated with severe joint lesions and extra-articular organ damage (2–4). Rheumatoid factor (RF) is an autoantibody that primarily targets the Fc fragment of IgG antibodies (5). Seropositive RA refers to rheumatoid arthritis where patients exhibit the presence of RF or ACPA, often involving early damage to lung tissue (6). Inflammation-related mechanisms in the lungs are crucial in the onset and progression of seropositive RA, involving the mucosal barrier hypothesis, neutrophil extracellular traps, pathogenic infections like COVID-19, Vitamin D, the microbiome and gut-lung axis, inhalation exposures and chronic pulmonary diseases (7–10).

Therefore, exploring the role of pulmonary inflammation-related biological mechanisms in the pathogenesis of seropositive RA holds significant clinical implications. This review explores how pulmonary inflammation influences the onset and progression of seropositive RA, offering insights and theoretical foundations for its early detection, diagnosis, and treatment.

## 2 Seropositive rheumatoid arthritis

The global prevalence of RA has remained approximately 0.46% over the last 40 years (11). RA is an autoimmune disease mainly characterized by synovitis, with pulmonary involvement frequently examined as an extra-articular manifestation (12). RA is categorized into seropositive and seronegative types based on the presence of RF and ACPA, showing notable differences in risk factors, clinical features, and prognosis (13). The diagnosis of seropositive RA relies on the detection of key biomarkers, such as RF and ACPA (14, 15).

However, many studies have shown that seropositive RA patients may exhibit RA-specific biomarkers in the blood for years without meeting the clinical or histological criteria for joint pathology (16–18). The occurrence of ACPA or RF serological markers prior to joint pathology suggests that joint involvement may not be the initial site of disease manifestation in seropositive RA patients. Although this hypothesis is yet to be systematically validated or supported by sufficient evidence, the possibility of pulmonary inflammatory lesions serving as the initial autoimmune site in seropositive RA is gaining increasing attention (19). Therefore, this study provides a comprehensive review of the potential link between pulmonary inflammation-related biological mechanisms and seropositive RA.

## 3 Biological mechanisms of pulmonary inflammation and seropositive rheumatoid arthritis

### 3.1 Mucosal barrier hypothesis

The large mucosal surface area of the lungs is exposed to the external environment, coming into direct contact with antigens, pollutants, and microorganisms, thereby maintaining a relatively active immune response over time (20). Recurrent lung inflammation can persistently activate mucosal immunity, potentially resulting in abnormal protein and autoantibody production, which may progress to affect the synovial tissue. In studies on the preclinical stage of seropositive RA, serum samples from high-risk individuals show elevated inflammatory factors, altered T-cell phenotypes, and expanded autoantibody profiles (7, 21–28). Additionally, the serum biomarker patterns in high-risk individuals were consistent with those found in retrospective studies of RA patients (7). Therefore, studying the origin of these biomarkers in high-risk populations for seropositive RA is of great significance, as pulmonary mucosal inflammation may be one of the causes of immune abnormalities in these individuals. Research on mucosal immunity indicates that IgA-ACPA and RF emerge long before the onset of joint symptoms in seropositive RA. The production of IgA isotypes ACPA and RF is common in mucosal tissues and has been shown to be associated with local mucosal inflammation (29–31). Through sequencing and characterization of plasmablasts in high-risk individuals, control subjects, and early seropositive RA patients, IgA and IgG antibodies have been phylogenetically shown to have a close relationship (32–34). The compromised mucosal barrier integrity can lead to antibodies entering the systemic circulation (35). This indicates that in seropositive RA, a persistent immune response linked to mucosal antigens might occur, with mucosal barrier dysfunction and systemic spread of IgG autoantibodies potentially being crucial early events in preclinical development.

### 3.2 Neutrophil extracellular traps

Neutrophil extracellular traps (NETs) are web-like structures composed of DNA and neutrophil-derived proteins that can rapidly control infections and exert immune functions, inducing local inflammation and tissue damage (36). The persistent activation of neutrophils and the formation and scaffolding role of NETs are closely associated with the local production of IgA ACPA in the lungs (37). During pulmonary infections, inflammatory mediators released at the infection site, together with locally produced ACPA, can trigger the formation of NETs through a process known as NETosis. Notably, in patients with RA-associated pulmonary inflammation, neutrophils demonstrate a markedly increased ability to form NETs (38). Growing evidence indicates that chronic airway inflammation contributes to the onset of seropositive RA by promoting NETosis, which leads to the breakdown of immune tolerance (39). Furthermore, the citrullination of various proteins within NETs has been identified as a critical factor driving ACPA production during the progression of RA (40). This process further amplifies NETosis by activating inflammasomes in macrophages, thereby promoting an immunogenic and pro-inflammatory microenvironment (41). The generation of ACPA and the persistence of inflammatory conditions are key mechanisms that drive the onset and progression of seropositive RA. During NET formation or apoptosis, elevated intracellular calcium concentrations lead to Ca²^+^ binding to the Ca²^+^-binding sites of peptidylarginine deiminases (PADs), resulting in their conformational activation (42). Activation of PADs enhances ACPA production, thereby contributing to the autoimmune pathogenesis of seropositive RA. The link between PAD activity and inflammatory states has been experimentally validated, with both factors working synergistically to drive the formation of NETs (43). Through these mechanisms, immune tolerance is compromised, establishing a self-perpetuating inflammatory state. The systemic spread of seropositive RA-associated immune cells, autoantibodies (particularly ACPA), and inflammatory mediators via the bloodstream constitutes a crucial pathway that drives both the onset of immune dysregulation and the ongoing inflammation characteristic of seropositive RA. Disruption of this process can exacerbate inflammation and tissue damage, creating a vicious cycle, and the notable inhibitory effects of specific PAD4 inhibitors on protein citrullination and NET formation provide supporting evidence for this hypothesis (43, 44).

The body has mechanisms to limit this vicious cycle. Myeloid inhibitory C-type lectin-like receptor (MICL) is predominantly found on myeloid cell surfaces, functioning as an inhibitory receptor. It regulates local immune responses, particularly the inflammatory response during cell death (45–47). The study by Malamud et al. demonstrated that MICL can recognize DNA within NETs and regulate NET formation by inhibiting neutrophil activation. Deficiency or inhibition of MICL triggers excessive NET formation via the ROS-PAD4 pathway, establishing a pathogenic autoinflammatory feedback loop in seropositive RA. They also confirmed a significant increase in anti-MICL autoantibody titers in the early stages of seropositive RA, with a correlation between anti-MICL antibody levels and ACPA levels (48). Therefore, anti-MICL antibodies produced in seropositive RA patients may be one of the factors that promote disease onset. When NETs are released, the resulting citrullinated neoepitopes promote the production of ACPA. Loss or inhibition of MICL function increases NET formation, leading to the development of a positive feedback loop in autoimmunity (49).

Furthermore, abnormal expression of the protein tyrosine phosphatase, non-receptor type 22 (PTPN22) gene leading to hypercitrullination of proteins can increase the propensity for NET formation, which is not uncommon in seropositive RA patients (50, 51). PTPN22 is a phosphatase involved in regulating immune responses and can also inhibit the process of histone citrullination (52). In peripheral CD4^+^ T cells, dysfunction of the PTPN22 gene may lead to abnormal protein citrullination, elevated Th17 cytokines, and decreased Th2 cytokines (52). This indicates that CD4^+^ T cells with dysfunctional PTPN22 are more prone to inflammation, aligning with mucosal inflammation and potentially linked to ACPA production.

### 3.3 Pathogenic microbial infections and COVID-19

**Figure 1:**
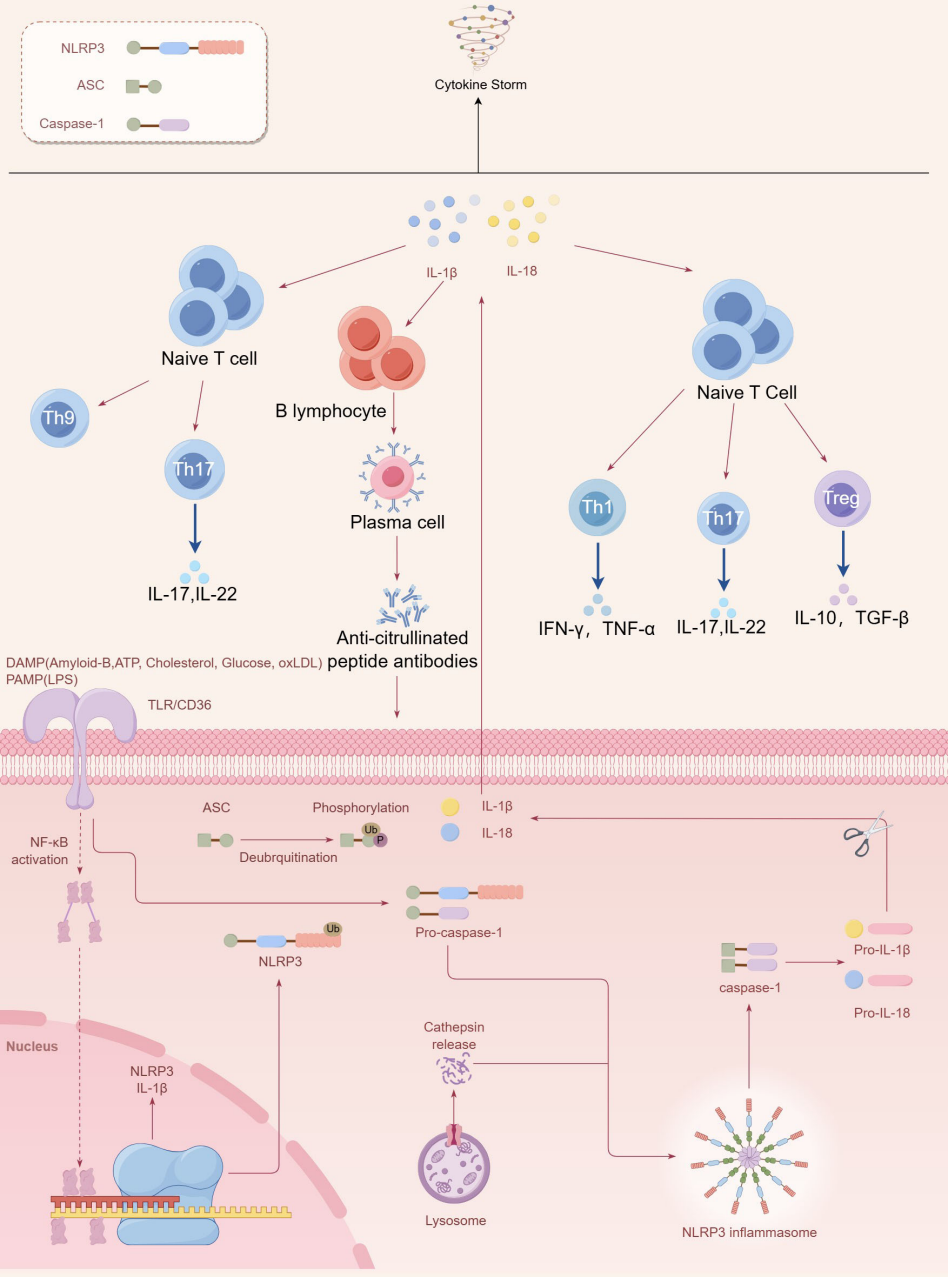
The vicious cycle in the lungs of seropositive rheumatoid arthritis patients. Inflammation triggers the NF-κB signaling pathway via pathogen-associated molecular pattern (PAMP) or damage-associated molecular pattern (DAMP) receptors, leading to the expression of NLRP3 and IL-1β. NLRP3 proteins assemble into the NLRP3 inflammasome complex, activating caspase-1 to trigger pyroptosis and facilitate the maturation of IL-1β and IL-18, thereby initiating downstream immune responses. The NLRP3-mediated inflammatory response can ultimately promote B cell proliferation and antibody production as well as T cell proliferation, differentiation, and cytokine release. ACPA enhances the expression of NLRP3 and IL-1β. This abnormal activation may create a vicious cycle in the lungs of seropositive RA patients and potentially lead to a cytokine storm.

The lungs are a common site for pathogenic microbial infections that cause tissue damage and trigger inflammation. During the inflammatory process, the pattern recognition receptor NOD-like receptor protein 3 (NLRP3) can detect and respond to pathogenic stimuli, assembling the NLRP3 inflammasome and activating caspase-1, which induces pyroptosis and promotes the maturation of interleukin-1 (IL-1β) and IL-18, thereby triggering an inflammatory response (53). This mechanism is particularly relevant in infections with certain serotypes of *Streptococcus pneumoniae* (54). Excessive activation of NLRP3 may be associated with the pathogenesis of seropositive RA. IL-1β is a key pro-inflammatory factor that facilitates inflammation by recruiting leukocytes, promoting systemic inflammation, inducing IL-12 to boost interferon-γ (IFN-γ) production, driving Th17 or Th9 cell differentiation, stimulating naive CD8^+^ T cell proliferation, differentiation, and migration, and promoting the proliferation of B cells and the synthesis of antibodies (55–62). IL-18 can direct Th1 responses and induce the production of IFN-γ and IL-8 (63–67). IL-18 originating from epithelial cells can also modulate the differentiation of Th17 cells and the functionality of regulatory T cells (Tregs). Moreover, a dysregulation in the Th17/Treg balance has been implicated in the pathogenesis of seropositive RA (66, 67). ACPA found in seropositive RA patients can enhance the expression of NLRP3 and IL-1β. This aberrant activation may establish a deleterious feedback loop in the lungs of seropositive RA patients, aggravating the disease condition (**Figure 1**) (68).

In addition, cytokines can play a significant role in the pathogenesis and progression of seropositive RA through other pathways. IL-1β can activate C/EBPβ, which, either alone or in synergy with NF-κB, upregulates the expression of factors such as tumor necrosis factor α (TNF-α), IL-1β, and IL-6, thereby exacerbating the inflammatory response in seropositive RA (69). IL-6 is widely involved in immune regulation and inflammatory responses and is a key factor in cytokine storms. Overproduction and unregulated receptor signaling may contribute to inflammatory diseases (70). IL-6 can influence the autoimmune response in seropositive RA by promoting Th17/Treg imbalance and can synergize with IL-21 to mediate antibody production, further exacerbating autoantibody production in seropositive RA (71, 72).

The massive release of cytokines triggered by pathogenic microbes may also cause immune dysregulation in other ways, inducing the onset of seropositive RA autoimmunity. Jonsson et al. (73) identified a novel population of CD8^+^ T cells with reduced cytotoxicity that can be rapidly activated by cytokines and produce high concentrations of IFN-γ and TNF-α, playing a key role in driving inflammation. These cells exhibit clonal expansion in inflamed tissues and can be activated both by cytokines such as IL-12 and in an antigen-specific manner, potentially linked to NLRP3 inflammasome-mediated inflammatory responses. This population is present in the blood, can migrate between tissues without changing phenotype, and shows higher enrichment in inflamed tissues. The characteristics of this novel CD8^+^ T cell population suggest that it may be one of the mechanisms by which pulmonary inflammation contributes to seropositive RA.

Cytokines may also play a crucial role in the pathogenesis of seropositive RA through the action of macrophages. Simmons et al. (74) demonstrated that signaling lymphocytic activation molecule family member 7 (SLAMF7) a receptor associated with macrophage hyperactivation in RA, and confirmed the presence of a SLAMF7 hyperactivated macrophage population in the synovial fluid of seropositive RA patients and in the lung tissue of COVID-19 patients. IFN-γ is a key regulator of SLAMF7 expression, driving robust expression of SLAMF7-mediated inflammatory factors and inducing a TNF-α autocrine signaling loop that amplifies inflammation. The novel CD8^+^ T cell population may contribute to the activation of this pathway by secreting large amounts of IFN-γ. This finding suggests a significant impact of COVID-19 on RA, though the relationship between the two requires further elucidation. Previous research indicates that TNF-α, a pro-inflammatory factor, contributes to the development of seropositive RA. However, it can also induce the expression of IL-7R on monocytes in the blood of seropositive RA patients, conferring anti-inflammatory properties on these monocytes (75). The imbalance between macrophage-mediated inflammatory and anti-inflammatory responses in RA patients may be one of the factors contributing to the disease’s onset and progression.

The COVID-19 pandemic has profoundly affected human health. During the COVID-19 pandemic, the overall incidence of RA increased significantly, particularly for seropositive RA (IRR = 1.60), suggesting an association between COVID-19 and seropositive RA (76). SARS-CoV-2, the virus responsible for COVID-19, can trigger autoimmune responses via inflammation and immune reactions, utilizing mechanisms like epitope spreading, molecular mimicry, and bystander activation (77). During the immune response to viral infection, interferon (IFN) is a key cytokine in antiviral immunity. Autoantibodies against IFN were detected in 13.7% of severe COVID-19 patients, indicating immune dysregulation in the context of SARS-CoV-2 infection (78). SARS-CoV-2 infection can also induce the generation of various autoantibodies. The acute phase is characterized by a severe inflammatory response, while the persistent inflammatory response observed during the recovery phase is associated with multiple autoimmune diseases (79).

COVID-19 primarily affects the lungs and can trigger excessive immune responses and cytokine storms, leading to severe pneumonia (80). Overproduction of factors like IL-6, IL-1β, and TNF mirrors the inflammatory response observed in RA. COVID-19 and seropositive RA both involve abnormal immune cell activation, resulting in tissue damage and inflammatory cell infiltration (81). SARS-CoV-2 infection can induce excessive inflammatory responses in seropositive RA patients by activating the NLRP3/CASP1 pyroptosis pathway and may enhance autoantibody production through similar mechanisms (82). In addition to the inflammatory response induced by innate immunity, an alternative and extrafollicular immune response to SARS-CoV-2 occurs in pulmonary tissues, which alters humoral immunity and memory T cell responses (83). This ultimately leads to breakdown of immune tolerance and generation of seropositive RA-associated autoantibodies. Studies have confirmed that the spike glycoprotein of SARS-CoV-2 shares 13 of 24 pentapeptides with pulmonary surfactant protein, which may contribute to specific immune responses and cross-reactivity following SARS-CoV-2 infection, potentially triggering seropositive RA (84). Moreover, studies demonstrate that impaired NET degradation in severe COVID-19 establishes a vicious cycle of NETosis and ACPA production, perpetuating a pro-inflammatory milieu in seropositive RA patients (85). Prolonged immune activation from COVID-19 may result in persistent autoantibodies, potentially causing seropositive RA. Additionally, SARS-CoV-2 infection may ultimately lead to pulmonary fibrosis, sharing similar mechanisms with RA-associated interstitial lung disease (RA-ILD), which will be discussed further (86).

### 3.4 Vitamin D

Vitamin D is crucial for infection resistance, inflammation reduction, and immune balance regulation (87). Vitamin D deficiency may exacerbate the burden of autoimmune diseases, a concern that is particularly pronounced during the COVID-19 pandemic (88). Vitamin D requires activation within the body to exert its physiological functions, and various immune cells can express vitamin D receptors and activating enzymes (89). 1,25-Dihydroxyvitamin D, the active form of vitamin D, promotes Th2 differentiation and the secretion of anti-inflammatory cytokines while reducing Th1 differentiation and the secretion of pro-inflammatory cytokines (such as IL-2, IFN-γ, and TNF-α). It also regulates the Th17/Treg balance and can inhibit the differentiation or maturation of naive B cells, indicating its potential to downregulate the activity of seropositive RA (90–92). Research on early RA patients indicates that vitamin D deficiency correlates with disease activity and serves as a predictive biomarker for disability progression within a year (93). Therefore, vitamin D potentially regulates pulmonary inflammation and may slow the progression of seropositive RA.

### 3.5 Microbiome and the gut-lung axis

The human microbiome, comprising all microorganisms residing in body cavities and on surfaces, impacts the host’s immune system via microbial antigens and metabolic products (94, 95). It is crucial for sustaining homeostasis and supporting immune function (96). Previously considered sterile, the lungs are the largest organ directly interacting with the environment (10, 97). Recent studies have identified the presence of bacteria in human lungs, noting their alterations in lung diseases and their links to alveolar immunity and disease outcomes (98, 99). The lung microbiome’s composition is mainly shaped by three factors: (i) the microbiota from the oral cavity, stomach, and air; (ii) mucociliary escalator and cough reflex clearance mechanisms; (iii) the local physicochemical conditions within the lungs (100). When lung inflammation occurs, the balance of these three factors may shift, potentially leading to alterations in the microbiome.

Pulmonary microbiota typically develops in areas of bronchial alteration, with a composition similar to that of the oropharynx but with a lower bacterial load (101). The pulmonary microbiota may be influenced by various regulators, impacting lung health. Vitamin D deficiency is linked to alterations in microbial populations and is associated with bronchiectasis and bacterial colonization (102). Alterations in the lung microbiota could significantly influence the progression of seropositive RA. Research indicates that seropositive RA patients exhibit significantly reduced microbial diversity and population imbalances in bronchoalveolar lavage samples compared to healthy individuals. Additionally, the disease activity of seropositive RA is significantly positively correlated with *Micrococcus* and *Renibaterium* (103). Therefore, alterations in the pulmonary microbiota are associated with seropositive RA; however, further research is needed to establish a causal relationship between the two.

The gut and lungs share a degree of homology in tissue embryology, resulting in certain similarities in their mucosal structure and function (104). The potential anatomical connections between the gut and lungs, along with the complex microbiota-related pathways involved, further support the existence of the gut-lung axis (105). Research indicates that changes in gut microbiota can influence lung immune responses via mechanisms involving Treg cells, toll-like receptors (TLRs), and inflammatory factors (106). Therefore, disruption of the gut microbiota is associated with various pulmonary diseases, including chronic obstructive pulmonary disease (COPD), asthma, cystic fibrosis, and interstitial lung disease (ILD) (100, 105, 107). These diseases may have an important impact on the development and progression of seropositive RA, which will be discussed in the following section. RA. Structural changes in the gut microbiota, through direct or indirect effects, can impact the onset of seropositive RA by decreasing anti-inflammatory microbes and increasing pro-inflammatory microbes (108). Studies have confirmed that the number of short-chain fatty acid-producing bacteria is significantly reduced in RA patients, while oral colonizing bacteria are increased. This transition results in decreased short-chain fatty acid-related metabolites and elevated amino acid and carbohydrate metabolites. These alterations show a positive correlation with pro-inflammatory cytokines like IL-6 and a negative correlation with anti-inflammatory cytokines such as IL-12, potentially affecting pulmonary inflammation via the gut-lung axis (109).

Pulmonary infections or chronic inflammation may alter the microbiota in both the lungs and gut, impacting local mucosal immune responses through multiple pathways and ultimately influencing joints via systemic inflammation (110). In seropositive RA patients, changes in the microbiota may affect the severity of joint symptoms, thereby influencing the assessment of seropositive RA activity. Animal studies on RA often use a collagen-induced arthritis model. During the preparation of this model, arthritic mice exhibit dysbiosis of the gut microbiota, and transplanting fecal matter from these arthritic mice to germ-free mice accelerates the development of arthritis (111). Another experiment demonstrated that using broad-spectrum antibiotics to modulate the microbiota prior to arthritis induction can reduce disease severity, while administering antibiotics in the late stages of arthritis induction can almost completely suppress the arthritis (112). This suggests that the role of the microbiome in the preclinical and clinical development of RA is multifaceted, potentially influencing various stages of the autoimmune initiation process, thereby impacting the activity of RA.

Mucosal dysbiosis can also trigger persistent mucosal inflammation, activate local innate immunity, and drive the disruption of the mucosal barrier, leading to the translocation of bacterial DNA, endotoxins, and other substances into circulation, which subsequently induces the production of systemic IgG autoantibodies (113, 114). The alleviation of mucosal inflammation in the lungs, intestines, and other sites is associated with substances such as resolvins (115, 116). Resolvins and protectins belong to the bioactive mediators synthesized from omega-3 polyunsaturated fatty acids (n-3 PUFAs). They are capable of reducing cellular activation and inflammation mediated by immune complexes (117). Chronic mucosal inflammation can lead to significant depletion of n-3 PUFAs, causing reduced circulating levels in RA patients (118). In patients with RA, persistent mucosal inflammation can deplete n-3 PUFAs, resulting in lower circulating levels (119, 120). This suggests a potentially close association between local mucosal inflammation, autoantibody production, and n-3 PUFA levels. Excessive mucosal inflammation and n-3 PUFA deficiency may both be essential factors for the persistent progression of RA.

Moreover, the mucosal microbiome may influence the development of seropositive RA through additional mechanisms. Alterations in mucosal surface microorganisms can lead to the occurrence of cross-reactivity, partially supporting a causal relationship between mucosal exposure, dysbiosis, and the progression of high-risk individuals to active RA (121). In seropositive RA patients, antigens carried by certain organisms can cross-react with ACPA (122), which may represent a key mechanism driving and influencing disease progression. It is currently known that seropositive RA is strongly linked to periodontitis resulting from Porphyromonas gingivalis infection, with underlying mechanisms involving molecular mimicry and specific antibody formation (123, 124). The periodontal pathogen Porphyromonas gingivalis encodes a PAD enzyme that citrullinates both bacterial and host proteins (125). Furthermore, periodontal abscesses can trigger the release of NETs, which, along with the aforementioned factors, contribute to the detection of ACP in the gingival crevicular fluid. These findings support the association between periodontal disease and increased citrullination within the inflammatory environment, which in turn impacts systemic ACPA levels (126–128). Previous studies have also demonstrated a link between oral bacteremia and RA flare-ups (129). Silva et al. (130, 131) confirmed a positive correlation between the severity of periodontitis and RA disease activity, showing that treatment of periodontal disease in RA patients was associated with a decrease in ACPA levels. They further noted that this reduction in ACPA was observed only in patients with baseline IgG ACPA levels below 150 AU (P = 0.006). These results support the hypothesis that ACPA responses are triggered and sustained within the gingival mucosa, and given the anatomical and microbiological similarities between the lung and oropharyngeal mucosa, this process may also influence the development of pulmonary mucosal responses.

In addition to the influence of the oral microbiota, the gut microbiota may also drive the development of seropositive RA through its interference with the immune system. Research by Pianta et al. (132) indicates a significant association between *Prevotella* species and the onset of RA. *Prevotella* can induce Th1-type immune responses, promote the production of ACPA-associated IgA antibodies and *Prevotella* DNA-associated IgG antibodies, and facilitate systemic dissemination of *Prevotella* DNA through phagocyte-mediated mechanisms. In addition to phagocytes, lymphocyte subsets in the mucosa can also migrate to other sites via circulation (133). Therefore, the interactions between immune cells and the microbiome may not only manifest locally but also influence immune and autoimmune responses in other tissues. This could be a critical step in the immunological dysregulation of T cells and B cells, wherein their activation occurs locally, such as in the lungs or intestinal mucosa, subsequently triggering systemic immune responses.

The composition of the pulmonary mucosal microbiome remains under investigation and is of significant importance to the onset and progression of seropositive RA. The relationship between the mucosa, its associated microbiome, and seropositive RA warrants further exploration.

### 3.6 Inhalation exposure and chronic pulmonary diseases

Inhalation exposure’s potential contribution to RA development has been widely researched (134). Inflammatory responses induced by the inhalation of toxic substances can promote the accumulation and activation of pulmonary antigen-presenting cells, induce the expression and activation of PAD, and enhance protein citrullination in the lungs, ultimately leading to the production of ACPA (135). Smoking has been established as a significant risk factor for seropositive RA, playing a pivotal role in the initial formation of RF and ACPA (136). In addition, air pollution factors such as silica, biomass burning, and PM2.5 are associated with higher rates of ACPA positivity (137–139). Studies have shown that the relationship between air pollution and RA-associated autoimmunity differs between high-risk individuals and seropositive RA patients (140). It is possible that air pollution may contribute to the progression of RA by facilitating the transition of RA-associated autoantibodies from pulmonary inflammation to synovitis. Aspiration of gastric contents due to gastroesophageal reflux disease, alongside environmental factors, is a risk factor for seropositive RA (141).

Inhalation exposures, including smoking, are risk factors for seropositive RA and contribute to chronic inflammatory lung diseases (142, 143). This may be one of the reasons for the close relationship between RA and chronic inflammatory pulmonary diseases. Inhalation exposure-induced pulmonary injury causes chronic inflammation in the lungs, which sustains the immune system in a state of prolonged high stress, potentially triggering and advancing seropositive RA via mechanisms like reactive oxygen species-induced tissue damage, airway remodeling-induced chronic hypoxia, molecular mimicry-driven autoimmunity, and chronic infections that enhance joint-specific susceptibility (144). Clinical evidence supports the involvement of airway processes in seropositive RA, showing the frequent coexistence of persistent airway inflammation and ACPA positivity both before and during disease onset (19). This likely reflects a key mechanism underlying the breakdown of immune tolerance and the persistence of inflammation in patients with seropositive RA. Related diseases include ILD, COPD, bronchiectasis, asthma, and others.

Epidemiological studies have found a significantly increased prevalence of pulmonary diseases during the preclinical phase of seropositive RA, supporting the notion that pulmonary diseases may be a risk factor for the development of seropositive RA (145). During the diagnosis of seropositive RA, ACPA demonstrate high specificity. However, ACPA can also be detected in some patients with chronic pulmonary diseases who have not yet been diagnosed with RA. This group has a significantly increased likelihood of developing seropositive RA in the future (146). The detection of ACPA prior to the clinical diagnosis of RA may be related to mucosal immune responses triggered by pulmonary inflammation. Research by Kelmenson et al. (147) indicates that during the period surrounding the clinical diagnosis of seropositive RA, the positivity rate of IgA-ACPA significantly increases, while the positivity rate of IgG-ACPA remains stable. This phenomenon suggests that IgA-dominated mucosal immunity may drive the onset of seropositive RA. Studies on lung biopsies and bronchoalveolar lavage fluid from early seropositive RA cases have shown that ACPA produced in the lungs shares some similar characteristics with ACPA produced in the joints (148). Moreover, structural abnormalities and inflammatory manifestations in the lungs may be associated with this process (149). The induction of bronchus-associated lymphoid tissue is a common structural abnormality in chronic pulmonary inflammation and is frequently detected in patients with seropositive RA (148). This structure has been shown to be associated with the local production of RF and ACPA (150).

ILD is often discussed as a consequence of the progression of seropositive RA. However, research indicates that abnormal protein modifications and immune responses triggered by smoking or other lung injuries can lead to ILD, which may subsequently induce secondary joint diseases, including seropositive RA (151). This indicates that the lungs might be the first location for immune tolerance disruption, with ILD possibly being the initial irregularity in seropositive RA development. However, ILD does not necessarily progress to RA. Multiple lines of evidence indicate distinct inflammatory components in the progression of RA-ILD compared to non-RA-related ILD. For instance, the influx of CXCR3-positive lymphocytes suggests a high local concentration of IFN-γ and the activation of Th1-type immune responses (152). Unlike non-RA-related ILD, the development of RA-ILD involves factors closely related to RA itself, such as abnormal activity of PAD, which is strongly associated with the production of autoantibodies, including ACPA (153). Research by Brink et al. (154) has demonstrated that ACPA is an independent risk factor for RA-ILD, with 11 types of ACPA targeting different antigens being associated with the development of pulmonary fibrosis.

COPD patients show increased pulmonary citrullination and a greater likelihood of producing diverse autoantibodies compared to those without airway diseases. This may increase susceptibility to seropositive RA (155, 156). Previous studies have indicated a significant association between seropositive RA and an increased risk of subsequently developing COPD (157). Therefore, small airway disease preceding the clinical diagnosis of COPD may be a contributing factor to the onset of seropositive RA. Evaluations performed on patients with similar small airway changes identified through high-resolution CT, using induced sputum collection, have detected RA-related IgA and IgG autoantibodies in the lungs, even when serum ACPA or RF is undetectable (31). These autoantibodies have been demonstrated to originate locally (30). Therefore, COPD may play a facilitating role in the development of seropositive RA, although further research is required to substantiate this relationship.

Chronic bacterial infections in seropositive RA patients with bronchiectasis may elevate autoantibody levels, indicating bronchiectasis could contribute to seropositive RA progression (158). However, although bronchiectasis may precede the joint manifestations of RA, it is often regarded as a late complication due to the immunosuppressive state of seropositive RA patients, which predisposes them to recurrent infections (159). A study involving seropositive RA patients without a clinical diagnosis of arthritis provided evidence supporting the possibility that chronic pulmonary inflammation triggers immune dysregulation in seropositive RA. In this study, 14% of the patients had bronchiectasis, and 76% exhibited pulmonary abnormalities (160). These findings suggest that the lungs, under chronic inflammatory conditions, may serve as the initial site for the production of autoantibodies in seropositive RA. However, the precise correlation and underlying mechanisms require further investigation to be fully elucidated.

The impact of asthma on the development of RA has been a subject of debate (161–164). However, a recent study indicates a correlation between asthma and an increased risk of seropositive RA, particularly over an extended period preceding its onset (> 0–10 years) (165). The specific mechanisms by which asthma influences the onset and progression of seropositive RA remain to be further investigated.

### 3.7 Anti-citrullinated protein antibodies

**Figure 2:**
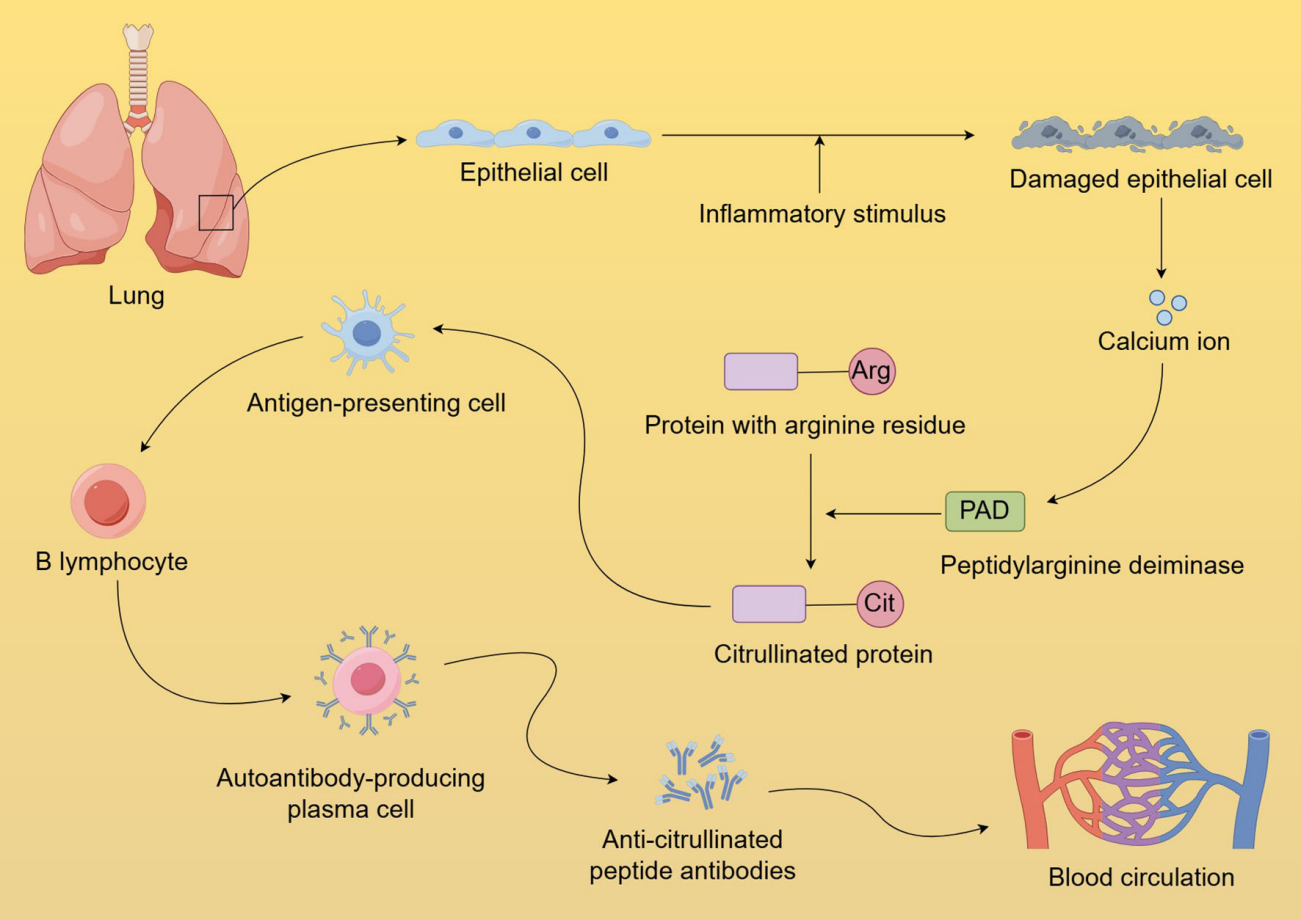
Formation and dissemination of anti-citrullinated protein antibodies in the lungs. Chronic pulmonary inflammation causes cellular damage and apoptosis, releasing significant calcium ions that activate PAD. PAD facilitates the transformation of protein arginine residues into citrulline, which disrupts immune tolerance, and triggers a specific immune response, resulting in ACPA production. The loss of mucosal barrier integrity caused by pulmonary inflammation further facilitates the leakage of abnormally produced ACPA into systemic circulation.

Extensive research has demonstrated that seropositive RA is closely associated with abnormal protein citrullination (166–168). Studies have found that ACPAs are present in the sputum of seropositive ACPA individuals without arthritis and are enriched in the bronchoalveolar lavage fluid of early ACPA-positive RA patients (31, 169). These phenomena suggest that pulmonary inflammation may play a role in the early stages of seropositive RA development. Repeated pulmonary inflammation leads to cellular damage and apoptosis, resulting in the release of large amounts of calcium ions, which activate PAD (170). PAD catalyzes the conversion of arginine residues in proteins to citrulline residues, leading to the deposition of citrullinated proteins in lung tissue. This disruption of immune tolerance leads to the presentation of citrullinated proteins as antigens by antigen-presenting cells, which triggers a specific immune response and the production of ACPAs (**Figure 2**) (171–174). The pulmonary inflammation-related biological mechanisms mentioned above may influence the onset of seropositive RA by triggering the extensive formation of ACPAs. Persistent pulmonary inflammation impacts mucosal immune responses through various pathways. In the progression from localized mucosal immune dysregulation to systemic seropositive RA, characterized predominantly by arthritis, several factors may play pivotal roles. These factors encompass shared antigenic targets between the lungs and joints, epitope spreading to joint-specific antigens, and the deposition of immune complexes within the joints (175).

## 4 The relationship between immune responses and seropositive rheumatoid arthritis inflammation

RA is a chronic, systemic autoimmune inflammatory disorder driven by complex, multifactorial interactions that involve dysregulation at various levels of the immune system (176). Specifically, the innate and adaptive immune systems form a coordinated yet dysregulated network that collectively drives both the onset and progression of RA-associated inflammation (177).

### 4.1 Innate immunity

Accumulating evidence highlights the role of innate immunity as both the initiator and perpetuator in the pathogenesis of seropositive RA. Innate immune components not only trigger the initial breach of self-tolerance but also actively sustain inflammatory cascades throughout disease progression (178). In addition to neutrophils and NETosis, the innate immune landscape in seropositive RA prominently involves monocytes, macrophages, and dendritic cells (DCs), which play critical roles in disease initiation and perpetuation through processes such as phagocytosis, antigen presentation, and cytokine production (179, 180).

Under homeostatic conditions, the maintenance of peripheral immune tolerance relies critically on immature dendritic cells (imDCs), which continuously sample and present self-antigens derived from tissue and blood (181, 182). Experimental evidence shows that depletion of imDCs leads to spontaneous autoimmunity, characterized by autoantibody production, inflammatory cell infiltration in peripheral organs, and a systemic wasting syndrome (183). The maturation of DCs is tightly regulated by TLR activation and influenced by various factors, including cytokines, hormones, vitamins, and environmental stimuli (178). During maturation, DCs undergo phenotypic reprogramming, marked by a downregulation of antigen uptake capacity and an upregulation of migration markers, which enables their trafficking to secondary lymphoid organs where they prime naïve T cells (184–186). Clinically, the induction of tolerogenic DCs in RA patients reduces activated T cell populations while increasing the Treg/effector T cell ratio, highlighting the important role of DCs in the pathogenesis of seropositive RA (187).

Research has shown that DCs in seropositive RA patients overproduce a wide range of immune mediators, including pro-inflammatory cytokines (such as IL-1, IL-6, IL-12, TNF, and IFN), differentiation factors (such as macrophage colony-stimulating factor and fibroblast growth factor), and chemokines (188–190). These DC-derived factors play key roles in sustaining synovial inflammation and are directly involved in several immunological processes that underpin RA pathogenesis (191). Activated by these inflammatory mediators, monocytes and macrophages not only amplify their own inflammatory responses but also become major producers of pathogenic cytokines, thereby driving a self-perpetuating cycle of inflammation and tissue destruction, which is central to RA pathogenesis and correlates with disease activity (192–196).

Innate lymphoid cells (ILCs) and natural killer (NK) cells play significant roles as regulators of inflammatory responses, contributing to the pathogenesis of autoimmunity in a dual manner (197). Studies have identified an altered ILC balance in RA patients, characterized by reduced frequencies of ILC2s in peripheral blood and synovial tissue, alongside an expansion of ILC3s. ILC2s exert protective effects through IL-9-mediated enhancement of Treg function, and their depletion is associated with increased arthritis severity (198–200). In contrast, ILC3s secrete IL-17 and other cytokines, which are now recognized as key mediators linking airway inflammation to RA pathogenesis (201–203). However, the precise role of NK cells in RA remains incompletely understood (204). Current evidence points to a compartmentalized dysregulation: activated NK cells accumulate in the synovium but are reduced in peripheral blood (205, 206). Synovial fluid analyses suggest that NK cells may exacerbate RA progression through two mechanisms: (i) secreting pro-inflammatory cytokines such as TNF-α and IFN-γ, and (ii) promoting osteoclastogenesis through cellular crosstalk (206, 207).

Collectively, pro-inflammatory cytokines (such as TNF-α and IFN-γ) released by activated innate immune cells drive the maturation of DCs, creating a self-amplifying loop that exacerbates innate immune activation and bridges to adaptive immunity, ultimately orchestrating the initiation and progression of seropositive RA.

### 4.2 Adaptive immunity

The breakdown of immune tolerance and the development of adaptive immunity-driven autoantibodies are hallmark features of seropositive RA. T lymphocytes and B lymphocytes, as key mediators of adaptive immunity, work synergistically with DCs to form a crucial link between innate and adaptive immune responses (208).

B cells play a central role in the pathogenesis of seropositive RA. Upon activation, they differentiate into autoantibody-producing plasma cells that generate RF and ACPAs, which are hallmarks of seropositive RA. In addition to their antibody-producing capacity, B cells contribute to disease progression through cytokine-mediated mechanisms, secreting pro-inflammatory factors including IL-1, IL-6, IL-8, IL-12, and TNF-α. These cytokines not only amplify immune dysregulation but also help establish and maintain a chronic inflammatory microenvironment that perpetuates disease activity (209, 210). B cell-derived cytokines also influence innate immune responses. For instance, TNF-α and IL-6 can activate synovial fibroblasts, while IL-8 promotes neutrophil infiltration——both mechanisms are closely associated with joint destruction in RA (211, 212). Additionally, IFN-γ produced during innate immune reactions can induce a significant increase in CXCR3^+^RANKL^+^ B cells in RA patients. These cells may further recruit IFN-γ-producing Th1 cells and additional CXCR3^+^RANKL^+^ B cells to the joint, establishing a pathogenic feedback loop that amplifies joint inflammation in RA (213, 214).

T cells play a significant role in the adaptive immune dysregulation of seropositive RA, particularly CD4^+^ T cells, which drive inflammation and support antibody production by B cells (215). The antigen-presenting function of innate immune cells, such as DCs and macrophages, along with B cells, is crucial for T cell activation (216). Upon activation, CD4^+^ T cells secrete pro-inflammatory cytokines, including IL-17, IL-21, granulocyte-macrophage colony-stimulating factor, TNF-α, IFN-γ, and CXCL13, which are essential for B cell recruitment and helper functions. These cytokines also modulate innate immune responses, representing a key mechanism in the initiation and perpetuation of seropositive RA (217–221). The precise role of CD8^+^ T cells in RA pathogenesis is still not fully understood. However, studies have shown that CD8^+^ T cells in RA patients exhibit significantly elevated IFN-γ production and are crucial for the formation of ectopic lymphoid neogenesis within the synovium, correlating with higher titers of RF and ACPAs (222–225).

Beyond the direct pro-inflammatory effects of adaptive immunity in RA, antibodies can also exert innate immune effector functions through their antigen-binding domains. These mechanisms include antibody-dependent cellular cytotoxicity, complement activation, and Fcγ receptor-mediated cell activation, all of which amplify inflammatory responses and contribute to joint damage (178).

As outlined above, innate and adaptive immunity do not function independently but rather work synergistically, creating a self-reinforcing autoimmune feedback loop. Pathogenic factors, both endogenous and exogenous, activate innate immune cells, triggering the release of inflammatory mediators that, in turn, activate the adaptive immune system. In response, activated adaptive immune cells exacerbate innate immune responses through cytokine production, further amplifying the inflammatory cascade and breaking immune tolerance. Simultaneously, the cytokines and other mediators produced by innate immunity actively regulate adaptive immune responses, likely contributing to the dysregulation of adaptive immunity and the destruction of adaptive immune cells. Collectively, the intra- and extra-articular inflammation characteristic of seropositive RA results from aberrant, mutually reinforcing activation of both immune arms. Understanding their interconnected regulatory mechanisms will not only reveal novel therapeutic targets but also provide a conceptual framework for future immune-modulatory strategies in seropositive RA.

## 5 Conclusions

In patients with seropositive RA, recurrent or chronic pulmonary inflammation may be associated with the disease’s onset through various biological mechanisms, underscoring the close relationship between pulmonary inflammatory factors and RA development. While seropositive RA is primarily characterized by joint symptoms, its systemic and multi-organ involvement requires greater emphasis. Among the extrajoint complications associated with seropositive RA, pulmonary diseases are relatively common and represent a significant cause of mortality in these patients. In certain cases, the progression of seropositive RA may originate from autoimmune processes in the lungs, manifest predominantly as joint symptoms, and culminate in severe pulmonary involvement during the disease’s terminal stages. The role of pulmonary immune dysregulation in the course of RA remains incompletely understood. The large volume and compensatory capacity of the lungs may obscure histological changes that precede joint symptoms, posing challenges for research into the mechanisms underlying seropositive RA. Inflammatory responses triggered by various pulmonary diseases need to be classified and studied in greater detail based on their specific immune characteristics to clarify their role in systemic immune dysregulation, thereby offering new avenues for the prevention and treatment of autoimmune diseases such as seropositive RA.

Studies have shown that the incidence of seropositive RA has been increasing. Early identification of seropositive RA is critical for reducing the disability associated with disease progression. Autoantibodies such as ACPA and RF can be detected prior to the clinical onset of seropositive RA, making them valuable for early diagnosis. As the close relationship between ACPA and pulmonary diseases becomes increasingly evident, screening and monitoring ACPA in individuals with long-standing pulmonary inflammation or seropositive RA patients without overt lung disease is of great significance. This may provide new hope for improving patients’ quality of life and extending their life expectancy. Additionally, further exploration of related biomarkers and their implications for systemic immunity is essential for advancing our understanding of seropositive RA progression and prognosis.

Thus, the prevention and early treatment of pulmonary inflammation should receive greater attention in the context of seropositive RA management. From the perspective of mitigating inflammation, maintaining a healthy microbiome, and supplementing n-3 PUFAs and vitamin D may serve as adjunctive measures for RA prevention and treatment. Recent studies have provided new directions for controlling inflammation in seropositive RA, including the suppression of inflammatory responses and the induction or enhancement of anti-inflammatory mechanisms. These strategies are particularly important for breaking the vicious cycles associated with seropositive RA pathogenesis. However, because these pathways are not unique to seropositive RA, they may present challenges in the development of targeted therapies. Further elucidation of the specific mechanisms underlying RA pathogenesis is needed to minimize potential adverse effects associated with new therapies.

## References

[1] Di Matteo A Bathon JM Emery P. Rheumatoid arthritis. Lancet. (2023) 402:2019–33. doi: 10.1016/S0140-6736(23)01525-8 38240831

[2] Mena-Vazquez N Perez AL Manrique-Arija S Romero BC Gomez CC Urena GI. Analysis of clinical-analytical characteristics in patients with rheumatoid arthritis and interstitial lung disease: case-control study. Reumatol Clin (Engl Ed). (2021) 17:197–202. doi: 10.1016/j.reuma.2019.06.001 31474500

[3] Liu YCLF. High levels of antibodies to citrullinated alpha-enolase peptide-1 (CEP-1) identify erosions and interstitial lung disease (ILD) in a Chinese rheumatoid arthritis cohort. Clin Immunol: Off J Clin Immunol Soc. (2019) 200:10–5. doi: 10.1016/j.clim.2019.01.001 30611755

[4] Giles JT Danoff SK Sokolove J Wagner CA Winchester R Pappas DA. Association of fine specificity and repertoire expansion of anticitrullinated peptide antibodies with rheumatoid arthritis associated interstitial lung disease. Ann Rheum Dis. (2014) 73:1487–94. doi: 10.1136/annrheumdis-2012-203160 PMC3883892 23716070

[5] Wu F Gao J Kang J Wang X Niu Q Liu J. B cells in rheumatoid arthritis:Pathogenic mechanisms and treatment prospects. Front Immunol. (2021) 12:750753. doi: 10.3389/fimmu.2021.750753 34650569 PMC8505880

[6] Malmstrom V Catrina AI Klareskog L. The immunopathogenesis of seropositive rheumatoid arthritis: from triggering to targeting. Nat Rev Immunol. (2017) 17:60–75. doi: 10.1038/nri.2016.124 27916980

[7] Holers VM Demoruelle MK Kuhn KA Buckner JH Robinson WH Okamoto Y. Rheumatoid arthritis and the mucosal origins hypothesis: protection turns to destruction. Nat Rev Rheumatol. (2018) 14:542–57. doi: 10.1038/s41584-018-0070-0 PMC6704378 30111803

[8] Hensvold AH Magnusson PK Joshua V Hansson M Israelsson L Ferreira R. Environmental and genetic factors in the development of anticitrullinated protein antibodies (ACPAs) and ACPA-positive rheumatoid arthritis: an epidemiological investigation in twins. Ann Rheum Dis. (2015) 74:375–80. doi: 10.1136/annrheumdis-2013-203947 24276366

[9] Khan T Jose RJ Renzoni EA Mouyis M. A closer look at the role of anti-CCP antibodies in the pathogenesis of rheumatoid arthritis-associated interstitial lung disease and bronchiectasis. Rheumatol Ther. (2021) 8:1463–75. doi: 10.1007/s40744-021-00362-4 PMC8572256 34449068

[10] Drakopanagiotakis F Stavropoulou E Tsigalou C Nena E Steiropoulos P. The role of the microbiome in connective-tissue-associated interstitial lung disease and pulmonary vasculitis. Biomedicines. (2022) 10:3195. doi: 10.3390/biomedicines10123195 36551951 PMC9775480

[11] Finckh A Gilbert B Hodkinson B Bae SC Thomas R Deane KD. Global epidemiology of rheumatoid arthritis. Nat Rev Rheumatol. (2022) 18:591–602. doi: 10.1038/s41584-022-00827-y 36068354

[12] Tanaka Y. Rheumatoid arthritis. Inflammation Regener. (2020) 40:20. doi: 10.1186/s41232-020-00133-8 PMC7487964 32944095

[13] Scott DL Wolfe F Huizinga TW. Rheumatoid arthritis. Lancet. (2010) 376:1094–108. doi: 10.1016/S0140-6736(10)60826-4 20870100

[14] Schellekens GA Visser H de Jong BA van den Hoogen FH Hazes JM Breedveld FC. The diagnostic properties of rheumatoid arthritis antibodies recognizing a cyclic citrullinated peptide. Arthritis Rheum. (2000) 43:155–63. doi: 10.1002/1529-0131(200001)43:1<155::AID-ANR20>3.0.CO;2-3 10643712

[15] Ronnelid J Turesson C Kastbom A. Autoantibodies in rheumatoid arthritis - laboratory and clinical perspectives. Front Immunol. (2021) 12:685312. doi: 10.3389/fimmu.2021.685312 34054878 PMC8161594

[16] Sokolove J Bromberg R Deane KD Lahey LJ Derber LA Chandra PE. Autoantibody epitope spreading in the pre-clinical phase predicts progression to rheumatoid arthritis. PloS One. (2012) 7:e35296. doi: 10.1371/journal.pone.0035296 22662108 PMC3360701

[17] Majka DS Deane KD Parrish LA Lazar AA Baron AE Walker CW. Duration of preclinical rheumatoid arthritis-related autoantibody positivity increases in subjects with older age at time of disease diagnosis. Ann Rheum Dis. (2008) 67:801–07. doi: 10.1136/ard.2007.076679 PMC3761074 17974596

[18] Rantapaa-Dahlqvist S de Jong BA Berglin E Hallmans G Wadell G Stenlund H. Antibodies against cyclic citrullinated peptide and IgA rheumatoid factor predict the development of rheumatoid arthritis. Arthritis Rheum. (2003) 48:2741–49. doi: 10.1002/art.11223 14558078

[19] Perry E Kelly C Eggleton P De Soyza A Hutchinson D. The lung in ACPA-positive rheumatoid arthritis: an initiating site of injury? Rheumatol (Oxford). (2014) 53:1940–50. doi: 10.1093/rheumatology/keu195 24831057

[20] Huff RD Carlsten C Hirota JA. An update on immunologic mechanisms in the respiratory mucosa in response to air pollutants. J Allergy Clin Immunol. (2019) 143:1989–2001. doi: 10.1016/j.jaci.2019.04.012 31176381

[21] Hunt L Hensor EM Nam J Burska AN Parmar R Emery P. T cell subsets: an immunological biomarker to predict progression to clinical arthritis in ACPA-positive individuals. Ann Rheum Dis. (2016) 75:1884–89. doi: 10.1136/annrheumdis-2015-207991 PMC5036223 27613874

[22] Young KA Deane KD Derber LA Hughes-Austin JM Wagner CA Sokolove J. Relatives without rheumatoid arthritis show reactivity to anti-citrullinated protein/peptide antibodies that are associated with arthritis-related traits: studies of the etiology of rheumatoid arthritis. Arthritis Rheum. (2013) 65:1995–2004. doi: 10.1002/art.38022 23754702 PMC3729718

[23] Hughes-Austin JM Deane KD Derber LA Kolfenbach JR Zerbe GO Sokolove J. Multiple cytokines and chemokines are associated with rheumatoid arthritis-related autoimmunity in first-degree relatives without rheumatoid arthritis: Studies of the Aetiology of Rheumatoid Arthritis (SERA). Ann Rheum Dis. (2013) 72:901–07. doi: 10.1136/annrheumdis-2012-201505 PMC3726193 22915618

[24] Arkema EV Goldstein BL Robinson W Sokolove J Wagner CA Malspeis S. Anti-citrullinated peptide autoantibodies, human leukocyte antigen shared epitope and risk of future rheumatoid arthritis: a nested case-control study. Arthritis Res Ther. (2013) 15:R159. doi: 10.1186/ar4342 24286474 PMC3953952

[25] El-Gabalawy HS Robinson DB Smolik I Hart D Elias B Wong K. Familial clustering of the serum cytokine profile in the relatives of rheumatoid arthritis patients. Arthritis Rheum. (2012) 64:1720–29. doi: 10.1002/art.34449 22354869

[26] van de Stadt LA van der Horst AR de Koning MH Bos WH Wolbink GJ van de Stadt RJ. The extent of the anti-citrullinated protein antibody repertoire is associated with arthritis development in patients with seropositive arthralgia. Ann Rheum Dis. (2011) 70:128–33. doi: 10.1136/ard.2010.132662 21062853

[27] Kolfenbach JR Deane KD Derber LA O’Donnell C Weisman MH Buckner JH. A prospective approach to investigating the natural history of preclinical rheumatoid arthritis (RA) using first-degree relatives of probands with RA. Arthritis Rheum. (2009) 61:1735–42. doi: 10.1002/art.24833 PMC2795101 19950324

[28] Nielen MM van Schaardenburg D Reesink HW Twisk JW van de Stadt RJ van der Horst-Bruinsma IE. Increased levels of C-reactive protein in serum from blood donors before the onset of rheumatoid arthritis. Arthritis Rheum. (2004) 50:2423–27. doi: 10.1002/art.20431 15334453

[29] Kokkonen H Mullazehi M Berglin E Hallmans G Wadell G Ronnelid J. Antibodies of IgG, IgA and IgM isotypes against cyclic citrullinated peptide precede the development of rheumatoid arthritis. Arthritis Res Ther. (2011) 13:R13. doi: 10.1186/ar3237 21291540 PMC3241357

[30] Demoruelle MK Harrall KK Ho L Purmalek MM Seto NL Rothfuss HM. Anti-citrullinated protein antibodies are associated with neutrophil extracellular traps in the sputum in relatives of rheumatoid arthritis patients. Arthritis Rheumatol. (2017) 69:1165–75. doi: 10.1002/art.40066 PMC5449213 28182854

[31] Willis VC Demoruelle MK Derber LA Chartier-Logan CJ Parish MC Pedraza IF. Sputum autoantibodies in patients with established rheumatoid arthritis and subjects at risk of future clinically apparent disease. Arthritis Rheum. (2013) 65:2545–54. doi: 10.1002/art.38066 PMC4066465 23817979

[32] Kinslow JD Blum LK Deane KD Demoruelle MK Okamoto Y Parish M. IgA plasmablasts are elevated in subjects at risk for future rheumatoid arthritis. Arthritis Rheumatol. (2016) 68:2372–83. doi: 10.1002/art.v68.10 PMC5042824 27273876

[33] Tan YC Blum LK Kongpachith S Ju CH Cai X Lindstrom TM. High-throughput sequencing of natively paired antibody chains provides evidence for original antigenic sin shaping the antibody response to influenza vaccination. Clin Immunol. (2014) 151:55–65. doi: 10.1016/j.clim.2013.12.008 24525048 PMC4006370

[34] Tan YC Kongpachith S Blum LK Ju CH Lahey LJ Lu DR. Barcode-enabled sequencing of plasmablast antibody repertoires in rheumatoid arthritis. Arthritis Rheumatol. (2014) 66:2706–15. doi: 10.1002/art.38754 PMC4560105 24965753

[35] Jorgensen C Moynier M Bologna C Youinou P Sany J. Rheumatoid factor associated with a secretory component in rheumatoid arthritis. Br J Rheumatol. (1995) 34:236–40. doi: 10.1093/rheumatology/34.3.236 7728398

[36] Drury B Hardisty G Gray RD Ho GT. Neutrophil extracellular traps in inflammatory bowel disease: pathogenic mechanisms and clinical translation. Cell Mol Gastroenterol Hepatol. (2021) 12:321–33. doi: 10.1016/j.jcmgh.2021.03.002 PMC8166923 33689803

[37] Wu S Peng W Liang X Wang W. Anti-citrullinated protein antibodies are associated with neutrophil extracellular trap formation in rheumatoid arthritis. J Clin Lab Anal. (2021) 35:e23662. doi: 10.1002/jcla.23662 33249645 PMC7957993

[38] Xue J Nian M Liang Y Zhu Z Hu Z Jia Y. Neutrophil extracellular traps (NETs) are increased in rheumatoid arthritis-associated interstitial lung disease. Respir Res. (2025) 26:33. doi: 10.1186/s12931-025-03111-1 39844268 PMC11756115

[39] Skopelja-Gardner S Jones JD Rigby WFC. NETtling” the host: Breaking of tolerance in chronic inflammation and chronic infection. J Autoimmun. (2018) 88:1–10. doi: 10.1016/j.jaut.2017.10.008 29100671

[40] Khandpur R Carmona-Rivera C Vivekanandan-Giri A Gizinski A Yalavarthi S Knight JS. NETs are a source of citrullinated autoantigens and stimulate inflammatory responses in rheumatoid arthritis. Sci Transl Med. (2013) 5:178ra40. doi: 10.1126/scitranslmed.3005580 PMC3727661 23536012

[41] Kahlenberg JM Carmona-Rivera C Smith CK Kaplan MJ. Neutrophil extracellular trap-associated protein activation of the NLRP3 inflammasome is enhanced in lupus macrophages. J Immunol. (2013) 190:1217–26. doi: 10.4049/jimmunol.1202388 PMC3552129 23267025

[42] Wang S Wang Y. Peptidylarginine deiminases in citrullination, gene regulation, health and pathogenesis. Biochim Biophys Acta. (2013) 1829:1126–35. doi: 10.1016/j.bbagrm.2013.07.003 PMC3775966 23860259

[43] Ciesielski O Biesiekierska M Panthu B Soszynski M Pirola L Balcerczyk A. Citrullination in the pathology of inflammatory and autoimmune disorders: recent advances and future perspectives. Cell Mol Life Sci. (2022) 79:94. doi: 10.1007/s00018-022-04126-3 35079870 PMC8788905

[44] Lewis HD Liddle J Coote JE Atkinson SJ Barker MD Bax BD. Inhibition of PAD4 activity is sufficient to disrupt mouse and human NET formation. Nat Chem Biol. (2015) 11:189–91. doi: 10.1038/nchembio.1735 PMC4397581 25622091

[45] Redelinghuys P Whitehead L Augello A Drummond RA Levesque JM Vautier S. MICL controls inflammation in rheumatoid arthritis. Ann Rheum Dis. (2016) 75:1386–91. doi: 10.1136/annrheumdis-2014-206644 PMC4941174 26275430

[46] Marshall AS Willment JA Pyz E Dennehy KM Reid DM Dri P. (CLEC12A) is differentially glycosylated and is down-regulated following cellular activation. Eur J Immunol. (2006) 36:2159–69. doi: 10.1002/eji.200535628 16838277

[47] Neumann K Castineiras-Vilarino M Hockendorf U Hannesschlager N Lemeer S Kupka D. Clec12a is an inhibitory receptor for uric acid crystals that regulates inflammation in response to cell death. Immunity. (2014) 40:389–99. doi: 10.1016/j.immuni.2013.12.015 24631154

[48] Malamud M Whitehead L McIntosh A Colella F Roelofs AJ Kusakabe T. Recognition and control of neutrophil extracellular trap formation by MICL. Nature. (2024) 633:442–50. doi: 10.1038/s41586-024-07820-3 PMC11390483 39143217

[49] Wigerblad G Kaplan MJ. Neutrophil extracellular traps in systemic autoimmune and autoinflammatory diseases. Nat Rev Immunol. (2023) 23:274–88. doi: 10.1038/s41577-022-00787-0 PMC9579530 36257987

[50] Chang HH Dwivedi N Nicholas AP Ho IC. The W620 polymorphism in PTPN22 disrupts its interaction with peptidylarginine deiminase type 4 and enhances citrullination and NETosis. Arthritis Rheumatol. (2015) 67:2323–34. doi: 10.1002/art.39215 26019128

[51] Ronninger M Guo Y Shchetynsky K Hill A Khademi M Olsson T. The balance of expression of PTPN22 splice forms is significantly different in rheumatoid arthritis patients compared with controls. Genome Med. (2012) 4:2. doi: 10.1186/gm301 22264340 PMC3334550

[52] Chang HH Liu GY Dwivedi N Sun B Okamoto Y Kinslow JD. A molecular signature of preclinical rheumatoid arthritis triggered by dysregulated PTPN22. JCI Insight. (2016) 1:e90045. doi: 10.1172/jci.insight.90045 27777982 PMC5070957

[53] Paik S Kim JK Silwal P Sasakawa C Jo EK. An update on the regulatory mechanisms of NLRP3 inflammasome activation. Cell Mol Immunol. (2021) 18:1141–60. doi: 10.1038/s41423-021-00670-3 PMC8093260 33850310

[54] Surabhi S Cuypers F Hammerschmidt S Siemens N. The role of NLRP3 inflammasome in pneumococcal infections. Front Immunol. (2020) 11:614801. doi: 10.3389/fimmu.2020.614801 33424869 PMC7793845

[55] Moorlag S Roring RJ Joosten L Netea MG. The role of the interleukin-1 family in trained immunity. Immunol Rev. (2018) 281:28–39. doi: 10.1111/imr.12617 29248003

[56] Chan AH Schroder K. Inflammasome signaling and regulation of interleukin-1 family cytokines. J Exp Med. (2020) 217:e20190314. doi: 10.1084/jem.20190314 31611248 PMC7037238

[57] Munoz-Wolf N Lavelle EC. A Guide to IL-1 family cytokines in adjuvanticity. FEBS J. (2018) 285:2377–401. doi: 10.1111/febs.14467 29656546

[58] Garlanda C Dinarello CA Mantovani A. The interleukin-1 family: back to the future. Immunity. (2013) 39:1003–18. doi: 10.1016/j.immuni.2013.11.010 PMC3933951 24332029

[59] Wilson NJ Boniface K Chan JR McKenzie BS Blumenschein WM Mattson JD. Development, cytokine profile and function of human interleukin 17-producing helper T cells. Nat Immunol. (2007) 8:950–57. doi: 10.1038/ni1497 17676044

[60] Xue G Jin G Fang J Lu Y. IL-4 together with IL-1beta induces antitumor Th9 cell differentiation in the absence of TGF-beta signaling. Nat Commun. (2019) 10:1376. doi: 10.1038/s41467-019-09401-9 30914642 PMC6435687

[61] Ben-Sasson SZ Wang K Cohen J Paul WE. IL-1beta strikingly enhances antigen-driven CD4 and CD8 T-cell responses. Cold Spring Harb Symp Quant Biol. (2013) 78:117–24. doi: 10.1101/sqb.2013.78.021246 24092469

[62] Ben-Sasson SZ Hogg A Hu-Li J Wingfield P Chen X Crank M. IL-1 enhances expansion, effector function, tissue localization, and memory response of antigen-specific CD8 T cells. J Exp Med. (2013) 210:491–502. doi: 10.1084/jem.20122006 23460726 PMC3600912

[63] Okamura H Nagata K Komatsu T Tanimoto T Nukata Y Tanabe F. A novel costimulatory factor for gamma interferon induction found in the livers of mice causes endotoxic shock. Infect Immun. (1995) 63:3966–72. doi: 10.1128/iai.63.10.3966-3972.1995 PMC173557 7558306

[64] Nakanishi K Yoshimoto T Tsutsui H Okamura H. Interleukin-18 is a unique cytokine that stimulates both Th1 and Th2 responses depending on its cytokine milieu. Cytokine Growth Factor Rev. (2001) 12:53–72. doi: 10.1016/s1359-6101(00)00015-0 11312119

[65] Poznanski SM Lee AJ Nham T Lusty E Larche MJ Lee DA. Combined stimulation with interleukin-18 and interleukin-12 potently induces interleukin-8 production by natural killer cells. J Innate Immun. (2017) 9:511–25. doi: 10.1159/000477172 PMC6738883 28633138

[66] Harrison OJ Srinivasan N Pott J Schiering C Krausgruber T Ilott NE. Epithelial-derived IL-18 regulates Th17 cell differentiation and Foxp3(+) Treg cell function in the intestine. Mucosal Immunol. (2015) 8:1226–36. doi: 10.1038/mi.2015.13 PMC4368110 25736457

[67] Li Z Guo J Bi L. Role of the NLRP3 inflammasome in autoimmune diseases. BioMed Pharmacother. (2020) 130:110542. doi: 10.1016/j.biopha.2020.110542 32738636

[68] Dong X Zheng Z Lin P Fu X Li F Jiang J. ACPAs promote IL-1beta production in rheumatoid arthritis by activating the NLRP3 inflammasome. Cell Mol Immunol. (2020) 17:261–71. doi: 10.1038/s41423-019-0201-9 PMC7052171 30911117

[69] Ren Q Liu Z Wu L Yin G Xie X Kong W. C/EBPbeta: The structure, regulation, and its roles in inflammation-related diseases. BioMed Pharmacother. (2023) 169:115938. doi: 10.1016/j.biopha.2023.115938 38000353

[70] Kang S Kishimoto T. Interplay between interleukin-6 signaling and the vascular endothelium in cytokine storms. Exp Mol Med. (2021) 53:1116–23. doi: 10.1038/s12276-021-00649-0 PMC8273570 34253862

[71] Grebenciucova E VanHaerents S. Interleukin 6: at the interface of human health and disease. Front Immunol. (2023) 14:1255533. doi: 10.3389/fimmu.2023.1255533 37841263 PMC10569068

[72] Kishimoto T Kang S. IL-6 revisited: from rheumatoid arthritis to CAR T cell therapy and COVID-19. Annu Rev Immunol. (2022) 40:323–48. doi: 10.1146/annurev-immunol-101220-023458 35113729

[73] Jonsson AH Zhang F Dunlap G Gomez-Rivas E Watts G Faust HJ. Granzyme K(+) CD8 T cells form a core population in inflamed human tissue. Sci Transl Med. (2022) 14:eabo0686. doi: 10.1126/scitranslmed.abo0686 35704599 PMC9972878

[74] Simmons DP Nguyen HN Gomez-Rivas E Jeong Y Jonsson AH Chen AF. SLAMF7 engagement superactivates macrophages in acute and chronic inflammation. Sci Immunol. (2022) 7:eabf2846. doi: 10.1126/sciimmunol.abf2846 35148199 PMC8991457

[75] Zhang B Zhang Y Xiong L Li Y Zhang Y Zhao J. CD127 imprints functional heterogeneity to diversify monocyte responses in inflammatory diseases. J Exp Med. (2022) 219:e20211191. doi: 10.1084/jem.20211191 35015026 PMC8757045

[76] Marin JS Mazenett-Granados EA Salazar-Uribe JC Sarmiento M Suarez JF Rojas M. Increased incidence of rheumatoid arthritis after COVID-19. Autoimmun Rev. (2023) 22:103409. doi: 10.1016/j.autrev.2023.103409 37597602

[77] Sundaresan B Shirafkan F Ripperger K Rattay K. The role of viral infections in the onset of autoimmune diseases. Viruses. (2023) 15:782. doi: 10.3390/v15030782 36992490 PMC10051805

[78] Moody R Wilson K Flanagan KL Jaworowski A Plebanski M. Adaptive immunity and the risk of autoreactivity in COVID-19. Int J Mol Sci. (2021) 22:8965. doi: 10.3390/ijms22168965 34445670 PMC8396528

[79] Rojas M Rodriguez Y Acosta-Ampudia Y Monsalve DM Zhu C Li QZ. Autoimmunity is a hallmark of post-COVID syndrome. J Transl Med. (2022) 20:129. doi: 10.1186/s12967-022-03328-4 35296346 PMC8924736

[80] Ehrenfeld M Tincani A Andreoli L Cattalini M Greenbaum A Kanduc D. Covid-19 and autoimmunity. Autoimmun Rev. (2020) 19:102597. doi: 10.1016/j.autrev.2020.102597 32535093 PMC7289100

[81] Schett G Manger B Simon D Caporali R. COVID-19 revisiting inflammatory pathways of arthritis. Nat Rev Rheumatol. (2020) 16:465–70. doi: 10.1038/s41584-020-0451-z PMC7304381 32561873

[82] Zheng Q Lin R Chen Y Lv Q Zhang J Zhai J. SARS-CoV-2 induces “cytokine storm” hyperinflammatory responses in RA patients through pyroptosis. Front Immunol. (2022) 13:1058884. doi: 10.3389/fimmu.2022.1058884 36532040 PMC9751040

[83] Larionova R Byvaltsev K Kravtsova COC Takha E Petrov S Kazarian G. SARS-Cov2 acute and post-active infection in the context of autoimmune and chronic inflammatory diseases. J Transl Autoimmun. (2022) 5:100154. doi: 10.1016/j.jtauto.2022.100154 35434592 PMC9005220

[84] Kanduc D Shoenfeld Y. On the molecular determinants of the SARS-CoV-2 attack. Clin Immunol. (2020) 215:108426. doi: 10.1016/j.clim.2020.108426 32311462 PMC7165084

[85] Garcia G Labrouche-Colomer S Duvignaud A Clequin E Dussiau C Tregouet D. Impaired balance between neutrophil extracellular trap formation and degradation by DNases in COVID-19 disease. J Transl Med. (2024) 22:246. doi: 10.1186/s12967-024-05044-7 38454482 PMC10919029

[86] Manfredi A Luppi F Cassone G Vacchi C Salvarani C Sebastiani M. Pathogenesis and treatment of idiopathic and rheumatoid arthritis-related interstitial pneumonia. The possible lesson from COVID-19 pneumonia. Expert Rev Clin Immunol. (2020) 16:751–70. doi: 10.1080/1744666X.2020.1803064 PMC7594185 32722946

[87] Ao T Kikuta J Ishii M. The effects of vitamin D on immune system and inflammatory diseases. Biomolecules. (2021) 11:1624. doi: 10.3390/biom11111624 34827621 PMC8615708

[88] Alobaid MA Alqabandi BS. SARS-CoV-2 induced vitamin D deficiency and psychological stress: a manifestation of autoimmune disease onset. Front Immunol. (2024) 15:1434486. doi: 10.3389/fimmu.2024.1434486 39416791 PMC11479920

[89] Hart PH Gorman S Finlay-Jones JJ. Modulation of the immune system by UV radiation: more than just the effects of vitamin D? Nat Rev Immunol. (2011) 11:584–96. doi: 10.1038/nri3045 21852793

[90] Skrobot A Demkow U Wachowska M. Immunomodulatory role of vitamin D: A review. Adv Exp Med Biol. (2018) 1108:13–23. doi: 10.1007/5584_2018_246 30143987

[91] Urry Z Chambers ES Xystrakis E Dimeloe S Richards DF Gabrysova L. The role of 1alpha,25-dihydroxyvitamin D3 and cytokines in the promotion of distinct Foxp3+ and IL-10+ CD4+ T cells. Eur J Immunol. (2012) 42:2697–708. doi: 10.1002/eji.201242370 PMC3471131 22903229

[92] Chen S Sims GP Chen XX Gu YY Chen S Lipsky PE. Modulatory effects of 1,25-dihydroxyvitamin D3 on human B cell differentiation. J Immunol. (2007) 179:1634–47. doi: 10.4049/jimmunol.179.3.1634 17641030

[93] Mouterde G Gamon E Rincheval N Lukas C Seror R Berenbaum F. Association between vitamin D deficiency and disease activity, disability, and radiographic progression in early rheumatoid arthritis: the ESPOIR cohort. J Rheumatol. (2020) 47:1624–28. doi: 10.3899/jrheum.190795 31839594

[94] La Barbera L Macaluso F Fasano S Grasso G Ciccia F Guggino G. Microbiome changes in connective tissue diseases and vasculitis: focus on metabolism and inflammation. Int J Mol Sci. (2022) 23:6532. doi: 10.3390/ijms23126532 35742974 PMC9224234

[95] Scher JU Littman R Abramson SB. Review: microbiome in inflammatory arthritis and human rheumatic diseases. Arthritis Rheumatol. (2016) 68:35–45. doi: 10.1002/art.39259 PMC4789258 26331579

[96] Longman RS Littman DR. The functional impact of the intestinal microbiome on mucosal immunity and systemic autoimmunity. Curr Opin Rheumatol. (2015) 27:381–87. doi: 10.1097/BOR.0000000000000190 PMC4929006 26002030

[97] Moffatt MF Cookson WO. The lung microbiome in health and disease. Clin Med (Lond). (2017) 17:525–29. doi: 10.7861/clinmedicine.17-6-525 PMC6297685 29196353

[98] Invernizzi R Lloyd CM Molyneaux PL. Respiratory microbiome and epithelial interactions shape immunity in the lungs. Immunology. (2020) 160:171–82. doi: 10.1111/imm.13195 PMC7218407 32196653

[99] Salisbury ML Han MK Dickson RP Molyneaux PL. Microbiome in interstitial lung disease: from pathogenesis to treatment target. Curr Opin Pulm Med. (2017) 23:404–10. doi: 10.1097/MCP.0000000000000399 PMC5638128 28650861

[100] Ntolios P Tzilas V Bouros E Avdoula E Karakasiliotis I Bouros D. The role of microbiome and virome in idiopathic pulmonary fibrosis. Biomedicines. (2021) 9:442. doi: 10.3390/biomedicines9040442 33924195 PMC8074588

[101] Venkataraman A Bassis CM Beck JM Young VB Curtis JL Huffnagle GB. Application of a neutral community model to assess structuring of the human lung microbiome. Mbio. (2015) 6:e02284-14. doi: 10.1128/mBio.02284-14 25604788 PMC4324308

[102] Ferri S Crimi C Heffler E Campisi R Noto A Crimi N. Vitamin D and disease severity in bronchiectasis. Respir Med. (2019) 148:1–05. doi: 10.1016/j.rmed.2019.01.009 30827468

[103] Scher JU Joshua V Artacho A Abdollahi-Roodsaz S Ockinger J Kullberg S. The lung microbiota in early rheumatoid arthritis and autoimmunity. Microbiome. (2016) 4:60. doi: 10.1186/s40168-016-0206-x 27855721 PMC5114783

[104] Marsland BJ Trompette A Gollwitzer ES. The gut-lung axis in respiratory disease. Ann Am Thorac Soc. (2015) 12 Suppl 2:S150–56. doi: 10.1513/AnnalsATS.201503-133AW 26595731

[105] Amati F Stainer A Mantero M Gramegna A Simonetta E Suigo G. Lung microbiome in idiopathic pulmonary fibrosis and other interstitial lung diseases. Int J Mol Sci. (2022) 23:977. doi: 10.3390/ijms23020977 35055163 PMC8779068

[106] McAleer JP Kolls JK. Contributions of the intestinal microbiome in lung immunity. Eur J Immunol. (2018) 48:39–49. doi: 10.1002/eji.201646721 28776643 PMC5762407

[107] Chioma OS Hesse LE Chapman A Drake WP. Role of the microbiome in interstitial lung diseases. Front Med (Lausanne). (2021) 8:595522. doi: 10.3389/fmed.2021.595522 33604346 PMC7885795

[108] Wang Y Wei J Zhang W Doherty M Zhang Y Xie H. Gut dysbiosis in rheumatic diseases: A systematic review and meta-analysis of 92 observational studies. EBioMedicine. (2022) 80:104055. doi: 10.1016/j.ebiom.2022.104055 35594658 PMC9120231

[109] Nagata N Takeuchi T Masuoka H Aoki R Ishikane M Iwamoto N. Human gut microbiota and its metabolites impact immune responses in COVID-19 and its complications. Gastroenterology. (2023) 164:272–88. doi: 10.1053/j.gastro.2022.09.024 PMC9499989 36155191

[110] Cao Z Li Q Wu J Li Y. Causal association of rheumatoid arthritis with obstructive lung disease: Evidence from Mendelian randomization study. Heart Lung. (2023) 62:35–42. doi: 10.1016/j.hrtlng.2023.05.020 37302263

[111] Liu X Zeng B Zhang J Li W Mou F Wang H. Role of the gut microbiome in modulating arthritis progression in mice. Sci Rep. (2016) 6:30594. doi: 10.1038/srep30594 27481047 PMC4969881

[112] Jubair WK Hendrickson JD Severs EL Schulz HM Adhikari S Ir D. Modulation of inflammatory arthritis in mice by gut microbiota through mucosal inflammation and autoantibody generation. Arthritis Rheumatol. (2018) 70:1220–33. doi: 10.1002/art.40490 PMC6105374 29534332

[113] Fuke N Nagata N Suganuma H Ota T. Regulation of gut microbiota and metabolic endotoxemia with dietary factors. Nutrients. (2019) 11:2277. doi: 10.3390/nu11102277 31547555 PMC6835897

[114] Zorgetto-Pinheiro VA Machate DJ Figueiredo PS Marcelino G Hiane PA Pott A. Omega-3 fatty acids and balanced gut microbiota on chronic inflammatory diseases: A close look at ulcerative colitis and rheumatoid arthritis pathogenesis. J Med Food. (2022) 25:341–54. doi: 10.1089/jmf.2021.0012 35438557

[115] Uddin M Levy BD. Resolvins: natural agonists for resolution of pulmonary inflammation. Prog Lipid Res. (2011) 50:75–88. doi: 10.1016/j.plipres.2010.09.002 20887750 PMC3012139

[116] Campbell EL Louis NA Tomassetti SE Canny GO Arita M Serhan CN. Resolvin E1 promotes mucosal surface clearance of neutrophils: a new paradigm for inflammatory resolution. FASEB J. (2007) 21:3162–70. doi: 10.1096/fj.07-8473com 17496159

[117] Souza PR Norling LV. Implications for eicosapentaenoic acid- and docosahexaenoic acid-derived resolvins as therapeutics for arthritis. Eur J Pharmacol. (2016) 785:165–73. doi: 10.1016/j.ejphar.2015.05.072 26165764

[118] Serhan CN. Pro-resolving lipid mediators are leads for resolution physiology. Nature. (2014) 510:92–101. doi: 10.1038/nature13479 24899309 PMC4263681

[119] Gan RW Demoruelle MK Deane KD Weisman MH Buckner JH Gregersen PK. Omega-3 fatty acids are associated with a lower prevalence of autoantibodies in shared epitope-positive subjects at risk for rheumatoid arthritis. Ann Rheum Dis. (2017) 76:147–52. doi: 10.1136/annrheumdis-2016-209154 PMC5371398 27190099

[120] Di Giuseppe D Wallin A Bottai M Askling J Wolk A. Long-term intake of dietary long-chain n-3 polyunsaturated fatty acids and risk of rheumatoid arthritis: a prospective cohort study of women. Ann Rheum Dis. (2014) 73:1949–53. doi: 10.1136/annrheumdis-2013-203338 23940215

[121] Li S Yu Y Yue Y Zhang Z Su K. Microbial infection and rheumatoid arthritis. J Clin Cell Immunol. (2013) 4:174. doi: 10.4172/2155-9899.1000174 25133066 PMC4131749

[122] Lundberg K Wegner N Yucel-Lindberg T Venables PJ. Periodontitis in RA-the citrullinated enolase connection. Nat Rev Rheumatol. (2010) 6:727–30. doi: 10.1038/nrrheum.2010.139 20820197

[123] Lopez-Oliva I Malcolm J Culshaw S. Periodontitis and rheumatoid arthritis-Global efforts to untangle two complex diseases. Periodontol 2000. (2024). doi: 10.1111/prd.12530 38411247

[124] Kapila YL. Oral health’s inextricable connection to systemic health: Special populations bring to bear multimodal relationships and factors connecting periodontal disease to systemic diseases and conditions. Periodontol 2000. (2021) 87:11–6. doi: 10.1111/prd.12398 PMC8457130 34463994

[125] Wegner N Wait R Sroka A Eick S Nguyen KA Lundberg K. Peptidylarginine deiminase from Porphyromonas gingivalis citrullinates human fibrinogen and alpha-enolase: implications for autoimmunity in rheumatoid arthritis. Arthritis Rheum. (2010) 62:2662–72. doi: 10.1002/art.27552 PMC2941529 20506214

[126] Eezammuddeen NN Vaithilingam RD Hassan N. Influence of periodontitis on levels of autoantibodies in rheumatoid arthritis patients: A systematic review. J Periodontal Res. (2023) 58:29–42. doi: 10.1111/jre.13065 36317493

[127] de Smit MJ Rahajoe PS Raveling-Eelsing E Lisotto P Harmsen H Kertia N. Influence of oral microbiota on the presence of IgA anti-citrullinated protein antibodies in gingival crevicular fluid. Front Health. (2022) 3:904711. doi: 10.3389/froh.2022.904711 PMC9243218 35784663

[128] Davison E Johnston W Piela K Rosier BT Paterson M Mira A. The subgingival plaque microbiome, systemic antibodies against bacteria and citrullinated proteins following periodontal therapy. Pathogens. (2021) 10:193. doi: 10.3390/pathogens10020193 33578802 PMC7916579

[129] Brewer RC Lanz TV Hale CR Sepich-Poore GD Martino C Swafford AD. Oral mucosal breaks trigger anti-citrullinated bacterial and human protein antibody responses in rheumatoid arthritis. Sci Transl Med. (2023) 15:eabq8476. doi: 10.1126/scitranslmed.abq8476 36812347 PMC10496947

[130] Silva DS De Vries C Rovisco J Serra S Kaminska M Mydel P. The impact of periodontitis and periodontal treatment on rheumatoid arthritis outcomes: an exploratory clinical trial. Rheumatol (Oxford). (2024) 64:1679–88. doi: 10.1093/rheumatology/keae358 39002123

[131] Silva DS Costa F Baptista IP Santiago T Lund H Tarp S. Evidence-based research on effectiveness of periodontal treatment in rheumatoid arthritis patients: A systematic review and meta-analysis. Arthritis Care Res (Hoboken). (2022) 74:1723–35. doi: 10.1002/acr.24622 33973383

[132] Pianta A Arvikar S Strle K Drouin EE Wang Q Costello CE. Evidence of the immune relevance of Prevotella copri, a gut microbe, in patients with rheumatoid arthritis. Arthritis Rheumatol. (2017) 69:964–75. doi: 10.1002/art.40003 PMC5406252 27863183

[133] Jacques P Elewaut D. Joint expedition: linking gut inflammation to arthritis. Mucosal Immunol. (2008) 1:364–71. doi: 10.1038/mi.2008.24 19079200

[134] Karlson EW Deane K. Environmental and gene-environment interactions and risk of rheumatoid arthritis. Rheum Dis Clin North Am. (2012) 38:405–26. doi: 10.1016/j.rdc.2012.04.002 PMC3402910 22819092

[135] Henrik K Bo D Leonid P Camilla B Johan R Lars K. Smoking is a major preventable risk factor for rheumatoid arthritis: estimations of risks after various exposures to cigarette smoke. Ann Rheum Dis. (2018) 70:508–11. 10.1136/ard.2009.120899 PMC3033966 21149499

[136] Kronzer VL Huang W Dellaripa PF Huang S Feathers V Lu B. Lifestyle and clinical risk factors for incident rheumatoid arthritis-associated interstitial lung disease. J Rheumatol. (2021) 48:656–63. doi: 10.3899/jrheum.200863 PMC8096643 33191286

[137] Ebel AV Lutt G Poole JA Thiele GM Baker JF Cannon GW. Association of agricultural, occupational, and military inhalants with autoantibodies and disease features in US veterans with rheumatoid arthritis. Arthritis Rheumatol. (2021) 73:392–400. doi: 10.1002/art.41559 33058561 PMC8236239

[138] Zhao N Al-Aly Z Zheng B van Donkelaar A Martin RV Pineau CA. Fine particulate matter components and interstitial lung disease in rheumatoid arthritis. Eur Respir J. (2022) 60:2102149. doi: 10.1183/13993003.02149-2021 34949700

[139] Too CL Muhamad NA Ilar A Padyukov L Alfredsson L Klareskog L. Occupational exposure to textile dust increases the risk of rheumatoid arthritis: results from a Malaysian population-based case-control study. Ann Rheum Dis. (2016) 75:997–1002. doi: 10.1136/annrheumdis-2015-208278 26681695 PMC4893106

[140] Gan RW Deane KD Zerbe GO Demoruelle MK Weisman MH Buckner JH. Relationship between air pollution and positivity of RA-related autoantibodies in individuals without established RA: a report on SERA. Ann Rheum Dis. (2013) 72:2002–05. doi: 10.1136/annrheumdis-2012-202949 PMC3818364 23572338

[141] Lin HC Xirasagar S Lee CZ Huang CC Chen CH. The association between gastro-oesophageal reflux disease and subsequent rheumatoid arthritis occurrence: a nested case-control study from Taiwan. BMJ Open. (2017) 7:e016667. doi: 10.1136/bmjopen-2017-016667 PMC5702028 29151046

[142] Park B Koo SM An J Lee M Kang HY Qiao D. Genome-wide assessment of gene-by-smoking interactions in COPD. Sci Rep. (2018) 8:9319. doi: 10.1038/s41598-018-27463-5 29915320 PMC6006158

[143] Kim K Jiang X Cui J Lu B Costenbader KH Sparks JA. Interactions between amino acid-defined major histocompatibility complex class II variants and smoking in seropositive rheumatoid arthritis. Arthritis Rheumatol. (2015) 67:2611–23. doi: 10.1002/art.39228 PMC4581918 26098791

[144] Friedlander HM Ford JA Zaccardelli A Terrio AV Cho MH Sparks JA. Obstructive lung diseases and risk of rheumatoid arthritis. Expert Rev Clin Immunol. (2020) 16:37–50. doi: 10.1080/1744666X.2019.1698293 31774329 PMC6980732

[145] Nikiphorou E de Lusignan S Mallen C Roberts J Khavandi K Bedarida G. Prognostic value of comorbidity indices and lung diseases in early rheumatoid arthritis: a UK population-based study. Rheumatol (Oxford). (2020) 59:1296–305. doi: 10.1093/rheumatology/kez409 PMC7244778 31580449

[146] England BR Hershberger D. Management issues in rheumatoid arthritis-associated interstitial lung disease. Curr Opin Rheumatol. (2020) 32:255–63. doi: 10.1097/BOR.0000000000000703 PMC7331796 32141954

[147] Kelmenson LB Wagner BD McNair BK Frazer-Abel A Demoruelle MK Bergstedt DT. Timing of elevations of autoantibody isotypes prior to diagnosis of rheumatoid arthritis. Arthritis Rheumatol. (2020) 72:251–61. doi: 10.1002/art.41091 PMC6994340 31464042

[148] Reynisdottir G Olsen H Joshua V Engstrom M Forsslund H Karimi R. Signs of immune activation and local inflammation are present in the bronchial tissue of patients with untreated early rheumatoid arthritis. Ann Rheum Dis. (2016) 75:1722–27. doi: 10.1136/annrheumdis-2015-208216 26530319

[149] Ytterberg AJ Joshua V Reynisdottir G Tarasova NK Rutishauser D Ossipova E. Shared immunological targets in the lungs and joints of patients with rheumatoid arthritis: identification and validation. Ann Rheum Dis. (2015) 74:1772–77. doi: 10.1136/annrheumdis-2013-204912 24817415

[150] Rangel-Moreno J Hartson L Navarro C Gaxiola M Selman M Randall TD. Inducible bronchus-associated lymphoid tissue (iBALT) in patients with pulmonary complications of rheumatoid arthritis. J Clin Invest. (2006) 116:3183–94. doi: 10.1172/JCI28756 PMC1678820 17143328

[151] Sullivan DI Ascherman DP. Rheumatoid arthritis-associated interstitial lung disease (RA-ILD): update on prevalence, risk factors, pathogenesis, and therapy. Curr Rheumatol Rep. (2024) 26:431–49. doi: 10.1007/s11926-024-01155-8 39320427

[152] Gautam M Masood MJ Arooj S Mahmud ME Mukhtar MU. Rheumatoid arthritis related interstitial lung disease: patterns of high-resolution computed tomography. Cureus. (2020) 12:e6875. doi: 10.7759/cureus.6875 32181104 PMC7053681

[153] Nava-Quiroz KJ Rojas-Serrano J Perez-Rubio G Buendia-Roldan I Mejia M Fernandez-Lopez JC. Molecular factors in PAD2 (PADI2) and PAD4 (PADI4) are associated with interstitial lung disease susceptibility in rheumatoid arthritis patients. Cells. (2023) 12:2235. doi: 10.3390/cells12182235 37759458 PMC10527441

[154] Brink M Ljung L Hansson M Ronnelid J Holmdahl R Skriner K. Anti-citrullinated protein antibody specificities and pulmonary fibrosis in relation to genetic loci in early rheumatoid arthritis. Rheumatol (Oxford). (2022) 61:4985–90. doi: 10.1093/rheumatology/keac280 PMC9729003 35532073

[155] Lugli EB Correia RE Fischer R Lundberg K Bracke KR Montgomery AB. Expression of citrulline and homocitrulline residues in the lungs of non-smokers and smokers: implications for autoimmunity in rheumatoid arthritis. Arthritis Res Ther. (2015) 17:9. doi: 10.1186/s13075-015-0520-x 25600626 PMC4349479

[156] Packard TA Li QZ Cosgrove GP Bowler RP Cambier JC. COPD is associated with production of autoantibodies to a broad spectrum of self-antigens, correlative with disease phenotype. Immunol Res. (2013) 55:48–57. doi: 10.1007/s12026-012-8347-x 22941590 PMC3919062

[157] Sparks JA Lin TC Camargo CJ Barbhaiya M Tedeschi SK Costenbader KH. Rheumatoid arthritis and risk of chronic obstructive pulmonary disease or asthma among women: A marginal structural model analysis in the Nurses’ Health Study. Semin Arthritis Rheum. (2018) 47:639–48. doi: 10.1016/j.semarthrit.2017.09.005 PMC5857435 29037522

[158] Quirke A-M Perry E Cartwright A Kelly C De Soyza A Eggleton P. Bronchiectasis is a model for chronic bacterial infection inducing autoimmunity in rheumatoid arthritis. Arthritis Rheumatol. (2015) 67:2335–42. doi: 10.1002/art.39226 PMC4832289 26017630

[159] Shadick NA Fanta CH Weinblatt ME O’Donnell W Coblyn JS. Bronchiectasis. A late feature of severe rheumatoid arthritis. Med (Baltimore). (1994) 73:161–70. doi: 10.1097/00005792-199405000-00005 8190039

[160] Demoruelle MK Weisman MH Simonian PL Lynch DA Sachs PB Pedraza IF. Brief report: airways abnormalities and rheumatoid arthritis-related autoantibodies in subjects without arthritis: early injury or initiating site of autoimmunity? Arthritis Rheum. (2012) 64:1756–61. doi: 10.1002/art.34344 PMC3319006 22183986

[161] Hou YC Hu HY Liu IL Chang YT Wu CY. The risk of autoimmune connective tissue diseases in patients with atopy: A nationwide population-based cohort study. Allergy Asthma Proc. (2017) 38:383–89. doi: 10.2500/aap.2017.38.4071 28814359

[162] Lai NS Tsai TY Koo M Lu MC. Association of rheumatoid arthritis with allergic diseases: A nationwide population-based cohort study. Allergy Asthma Proc. (2015) 36:99–103. doi: 10.2500/aap.2015.36.3871 26314811

[163] Hemminki K Li X Sundquist J Sundquist K. Risk of asthma and autoimmune diseases and related conditions in patients hospitalized for obesity. Ann Med. (2012) 44:289–95. doi: 10.3109/07853890.2010.547515 21284531

[164] Tirosh A Mandel D Mimouni FB Zimlichman E Shochat T Kochba I. Autoimmune diseases in asthma. Ann Intern Med. (2006) 144:877–83. doi: 10.7326/0003-4819-144-12-200606200-00004 16785476

[165] Kronzer VL Huang W Crowson CS DavisIII JM Vassallo R Doyle TJ. Timing of sinusitis and other respiratory tract diseases and risk of rheumatoid arthritis. Semin Arthritis Rheum. (2022) 52:151937. doi: 10.1016/j.semarthrit.2021.11.008 35042150 PMC8820230

[166] Curran AM Girgis AA Jang Y Crawford JD Thomas MA Kawalerski R. Citrullination modulates antigen processing and presentation by revealing cryptic epitopes in rheumatoid arthritis. Nat Commun. (2023) 14:1061. doi: 10.1038/s41467-023-36620-y 36828807 PMC9958131

[167] Haro I Sanmarti R Gomara MJ. Implications of post-translational modifications in autoimmunity with emphasis on citrullination, homocitrullination and acetylation for the pathogenesis, diagnosis and prognosis of rheumatoid arthritis. Int J Mol Sci. (2022) 23:15803. doi: 10.3390/ijms232415803 36555449 PMC9781636

[168] van Venrooij WJ Pruijn GJ. How citrullination invaded rheumatoid arthritis research. Arthritis Res Ther. (2014) 16:103. doi: 10.1186/ar4458 24472574 PMC4061769

[169] Reynisdottir G Karimi R Joshua V Olsen H Hensvold AH Harju A. Structural changes and antibody enrichment in the lungs are early features of anti-citrullinated protein antibody-positive rheumatoid arthritis. Arthritis Rheumatol. (2014) 66:31–9. doi: 10.1002/art.38201 24449573

[170] Medzhitov R. Origin and physiological roles of inflammation. Nature. (2008) 454:428–35. doi: 10.1038/nature07201 18650913

[171] Diesler R Cottin V. Pulmonary fibrosis associated with rheumatoid arthritis: from pathophysiology to treatment strategies. Expert Rev Respir Med. (2022) 16:541–53. doi: 10.1080/17476348.2022.2089116 35695895

[172] Rogers GE Harding HWJ Llewellyn-Smith IJ. The origin of citrulline-containing proteins in the hair follicle and the chemical nature of trichohyalin, an intracellular precursor. BBA Protein Structure. (1977) 495:159–75. doi: 10.1016/0005-2795(77)90250-1 410454

[173] ROGERS GE. Occurrence of citrulline in proteins. Nature. (1962) 194:1149–51. doi: 10.1038/1941149a0 14493344

[174] Rogers GE Simmonds DH. Content of citrulline and other amino-acids in a protein of hair follicles. Nature. (1958) 182:186–87. doi: 10.1038/182186a0 13566234

[175] Demoruelle MK Wilson TM Deane KD. Lung inflammation in the pathogenesis of rheumatoid arthritis. Immunol Rev. (2020) 294:124–32. doi: 10.1111/imr.12842 32030763

[176] Firestein GS. Evolving concepts of rheumatoid arthritis. Nature. (2003) 423:356–61. doi: 10.1038/nature01661 12748655

[177] Cutolo M Campitiello R Gotelli E Soldano S. The role of M1/M2 macrophage polarization in rheumatoid arthritis synovitis. Front Immunol. (2022) 13:867260. doi: 10.3389/fimmu.2022.867260 35663975 PMC9161083

[178] Saferding V Bluml S. Innate immunity as the trigger of systemic autoimmune diseases. J Autoimmun. (2020) 110:102382. doi: 10.1016/j.jaut.2019.102382 31883831

[179] Narasimhan PB Marcovecchio P Hamers AAJ Hedrick CC. Nonclassical monocytes in health and disease. Annu Rev Immunol. (2019) 37:439–56. doi: 10.1146/annurev-immunol-042617-053119 31026415

[180] Edilova MI Akram A Abdul-Sater AA. Innate immunity drives pathogenesis of rheumatoid arthritis. BioMed J. (2021) 44:172–82. doi: 10.1016/j.bj.2020.06.010 PMC8178572 32798211

[181] Hawiger D Inaba K Dorsett Y Guo M Mahnke K Rivera M. Dendritic cells induce peripheral T cell unresponsiveness under steady state conditions *in vivo* . J Exp Med. (2001) 194:769–79. doi: 10.1084/jem.194.6.769 PMC2195961 11560993

[182] Scheinecker C McHugh R Shevach EM Germain RN. Constitutive presentation of a natural tissue autoantigen exclusively by dendritic cells in the draining lymph node. J Exp Med. (2002) 196:1079–90. doi: 10.1084/jem.20020991 PMC2194046 12391019

[183] Ohnmacht C Pullner A King SBS Drexler I Meier S Brocker T. Constitutive ablation of dendritic cells breaks self-tolerance of CD4 T cells and results in spontaneous fatal autoimmunity. J Exp Med. (2009) 206:549–59. doi: 10.1084/jem.20082394 PMC2699126 19237601

[184] Banchereau J Steinman RM. Dendritic cells and the control of immunity. Nature. (1998) 392:245–52. doi: 10.1038/32588 9521319

[185] Iwasaki A Medzhitov R. Control of adaptive immunity by the innate immune system. Nat Immunol. (2015) 16:343–53. doi: 10.1038/ni.3123 PMC4507498 25789684

[186] Inaba K Turley S Iyoda T Yamaide F Shimoyama S Reis E Sousa C. The formation of immunogenic major histocompatibility complex class II-peptide ligands in lysosomal compartments of dendritic cells is regulated by inflammatory stimuli. J Exp Med. (2000) 191:927–36. doi: 10.1084/jem.191.6.927 PMC2193115 10727455

[187] Benham H Nel HJ Law SC Mehdi AM Street S Ramnoruth N. Citrullinated peptide dendritic cell immunotherapy in HLA risk genotype-positive rheumatoid arthritis patients. Sci Transl Med. (2015) 7:290ra87. doi: 10.1126/scitranslmed.aaa9301 26041704

[188] Moret FM Hack CE van der Wurff-Jacobs KMG de Jager W Radstake TRDJ Lafeber FPJG. Intra-articular CD1c-expressing myeloid dendritic cells from rheumatoid arthritis patients express a unique set of T cell-attracting chemokines and spontaneously induce Th1, Th17 and Th2 cell activity. Arthritis Res Ther. (2013) 15:R155. doi: 10.1186/ar4338 24286358 PMC3979121

[189] Lebre MC Jongbloed SL Tas SW Smeets TJM McInnes IB Tak PP. Rheumatoid arthritis synovium contains two subsets of CD83-DC-LAMP- dendritic cells with distinct cytokine profiles. Am J Pathol. (2008) 172:940–50. doi: 10.2353/ajpath.2008.070703 PMC2276434 18292234

[190] Lande R Giacomini E Serafini B Rosicarelli B Sebastiani GD Minisola G. Characterization and recruitment of plasmacytoid dendritic cells in synovial fluid and tissue of patients with chronic inflammatory arthritis. J Immunol. (2004) 173:2815–24. doi: 10.4049/jimmunol.173.4.2815 15295000

[191] McInnes IB Buckley CD Isaacs JD. Cytokines in rheumatoid arthritis - shaping the immunological landscape. Nat Rev Rheumatol. (2016) 12:63–8. doi: 10.1038/nrrheum.2015.171 26656659

[192] Van Raemdonck K Umar S Palasiewicz K Volkov S Volin MV Arami S. CCL21/CCR7 signaling in macrophages promotes joint inflammation and Th17-mediated osteoclast formation in rheumatoid arthritis. Cell Mol Life Sci. (2020) 77:1387–99. doi: 10.1007/s00018-019-03235-w PMC10040247 31342120

[193] Siouti E Andreakos E. The many facets of macrophages in rheumatoid arthritis. Biochem Pharmacol. (2019) 165:152–69. doi: 10.1016/j.bcp.2019.03.029 30910693

[194] Ardura JA Rackov G Izquierdo E Alonso V Gortazar AR Escribese MM. Targeting macrophages: friends or foes in disease? Front Pharmacol. (2019) 10:1255. doi: 10.3389/fphar.2019.01255 31708781 PMC6819424

[195] Udalova IA Mantovani A Feldmann M. Macrophage heterogeneity in the context of rheumatoid arthritis. Nat Rev Rheumatol. (2016) 12:472–85. doi: 10.1038/nrrheum.2016.91 27383913

[196] Bluml S Redlich K Smolen JS. Mechanisms of tissue damage in arthritis. Semin Immunopathol. (2014) 36:531–40. doi: 10.1007/s00281-014-0442-8 25212687

[197] Xiong T Turner J. Innate lymphoid cells in autoimmunity and chronic inflammatory diseases. Semin Immunopathol. (2018) 40:393–406. doi: 10.1007/s00281-018-0670-4 29568972

[198] Omata Y Frech M Primbs T Lucas S Andreev D Scholtysek C. Group 2 innate lymphoid cells attenuate inflammatory arthritis and protect from bone destruction in mice. Cell Rep. (2018) 24:169–80. doi: 10.1016/j.celrep.2018.06.005 29972778

[199] Rauber S Luber M Weber S Maul L Soare A Wohlfahrt T. Resolution of inflammation by interleukin-9-producing type 2 innate lymphoid cells. Nat Med. (2017) 23:938–44. doi: 10.1038/nm.4373 PMC5575995 28714991

[200] Leijten EFA van Kempen TS Boes M Michels-van Amelsfort JMR Hijnen D Hartgring SAY. Brief report: enrichment of activated group 3 innate lymphoid cells in psoriatic arthritis synovial fluid. Arthritis Rheumatol. (2015) 67:2673–78. doi: 10.1002/art.39261 26137857

[201] Mannion JM McLoughlin RM Lalor SJ. The airway microbiome-IL-17 axis: a critical regulator of chronic inflammatory disease. Clin Rev Allergy Immunol. (2023) 64:161–78. doi: 10.1007/s12016-022-08928-y PMC10017631 35275333

[202] Berry SPD Dossou C Kashif A Sharifinejad N Azizi G Hamedifar H. The role of IL-17 and anti-IL-17 agents in the immunopathogenesis and management of autoimmune and inflammatory diseases. Int Immunopharmacol. (2022) 102:108402. doi: 10.1016/j.intimp.2021.108402 34863654

[203] Spits H Artis D Colonna M Diefenbach A Di Santo JP Eberl G. Innate lymphoid cells–a proposal for uniform nomenclature. Nat Rev Immunol. (2013) 13:145–49. doi: 10.1038/nri3365 23348417

[204] Kucuksezer UC Aktas Cetin E Esen F Tahrali I Akdeniz N Gelmez MY. The role of natural killer cells in autoimmune diseases. Front Immunol. (2021) 12:622306. doi: 10.3389/fimmu.2021.622306 33717125 PMC7947192

[205] Chalan P Bijzet J Kroesen B Boots AMH Brouwer E. Altered natural killer cell subsets in seropositive arthralgia and early rheumatoid arthritis are associated with autoantibody status. J Rheumatol. (2016) 43:1008–16. doi: 10.3899/jrheum.150644 27036380

[206] Aggarwal A Sharma A Bhatnagar A. Role of cytolytic impairment of natural killer and natural killer T-cell populations in rheumatoid arthritis. Clin Rheumatol. (2014) 33:1067–78. doi: 10.1007/s10067-014-2641-z 24797770

[207] Ahern DJ Brennan FM. The role of Natural Killer cells in the pathogenesis of rheumatoid arthritis: major contributors or essential homeostatic modulators? Immunol Lett. (2011) 136:115–21. doi: 10.1016/j.imlet.2010.11.001 21073898

[208] Gray KJ Gibbs JE. Adaptive immunity, chronic inflammation and the clock. Semin Immunopathol. (2022) 44:209–24. doi: 10.1007/s00281-022-00919-7 PMC8901482 35233691

[209] Cowan GJM Miles K Capitani L Giguere SSB Johnsson H Goodyear C. In human autoimmunity, a substantial component of the B cell repertoire consists of polyclonal, barely mutated IgG(+ve) B cells. Front Immunol. (2020) 11:395. doi: 10.3389/fimmu.2020.00395 32265907 PMC7099054

[210] Yeo L Toellner K Salmon M Filer A Buckley CD Raza K. Cytokine mRNA profiling identifies B cells as a major source of RANKL in rheumatoid arthritis. Ann Rheum Dis. (2011) 70:2022–28. doi: 10.1136/ard.2011.153312 PMC3184241 21742639

[211] Kristyanto H Blomberg NJ Slot LM van der Voort EIH Kerkman PF Bakker A. Persistently activated, proliferative memory autoreactive B cells promote inflammation in rheumatoid arthritis. Sci Transl Med. (2020) 12. doi: 10.1126/scitranslmed.aaz5327 PMC7615909 33208502

[212] Storch H Zimmermann B Resch B Tykocinski L Moradi B Horn P. Activated human B cells induce inflammatory fibroblasts with cartilage-destructive properties and become functionally suppressed in return. Ann Rheum Dis. (2016) 75:924–32. doi: 10.1136/annrheumdis-2014-206965 25985971

[213] Nakayama T Yoshimura M Higashioka K Miyawaki K Ota Y Ayano M. Type 1 helper T cells generate CXCL9/10-producing T-bet(+) effector B cells potentially involved in the pathogenesis of rheumatoid arthritis. Cell Immunol. (2021) 360:104263. doi: 10.1016/j.cellimm.2020.104263 33387686

[214] Ota Y Niiro H Ota S Ueki N Tsuzuki H Nakayama T. Generation mechanism of RANKL(+) effector memory B cells: relevance to the pathogenesis of rheumatoid arthritis. Arthritis Res Ther. (2016) 18:67. doi: 10.1186/s13075-016-0957-6 26980135 PMC4793760

[215] Kondo Y Yokosawa M Kaneko S Furuyama K Segawa S Tsuboi H. Review: transcriptional regulation of CD4+ T cell differentiation in experimentally induced arthritis and rheumatoid arthritis. Arthritis Rheumatol. (2018) 70:653–61. doi: 10.1002/art.40398 PMC5947164 29245178

[216] Takemura S Klimiuk PA Braun A Goronzy JJ Weyand CM. T cell activation in rheumatoid synovium is B cell dependent. J Immunol. (2001) 167:4710–18. doi: 10.4049/jimmunol.167.8.4710 11591802

[217] Sakuragi T Yamada H Haraguchi A Kai K Fukushi J Ikemura S. Autoreactivity of peripheral helper T cells in the joints of rheumatoid arthritis. J Immunol. (2021) 206:2045–51. doi: 10.4049/jimmunol.2000783 33846228

[218] Takeshita M Suzuki K Kondo Y Morita R Okuzono Y Koga K. Multi-dimensional analysis identified rheumatoid arthritis-driving pathway in human T cell. Ann Rheum Dis. (2019) 78:1346–56. doi: 10.1136/annrheumdis-2018-214885 PMC6788883 31167762

[219] Zhang F Wei K Slowikowski K Fonseka CY Rao DA Kelly S. Defining inflammatory cell states in rheumatoid arthritis joint synovial tissues by integrating single-cell transcriptomics and mass cytometry. Nat Immunol. (2019) 20:928–42. doi: 10.1038/s41590-019-0378-1 PMC6602051 31061532

[220] Reynolds G Gibbon JR Pratt AG Wood MJ Coady D Raftery G. Synovial CD4+ T-cell-derived GM-CSF supports the differentiation of an inflammatory dendritic cell population in rheumatoid arthritis. Ann Rheum Dis. (2016) 75:899–907. doi: 10.1136/annrheumdis-2014-206578 25923217 PMC4853576

[221] Harrington LE Hatton RD Mangan PR Turner H Murphy TL Murphy KM. Interleukin 17-producing CD4+ effector T cells develop via a lineage distinct from the T helper type 1 and 2 lineages. Nat Immunol. (2005) 6:1123–32. doi: 10.1038/ni1254 16200070

[222] Kang YM Zhang X Wagner UG Yang H Beckenbaugh RD Kurtin PJ. CD8 T cells are required for the formation of ectopic germinal centers in rheumatoid synovitis. J Exp Med. (2002) 195:1325–36. doi: 10.1084/jem.20011565 PMC2193749 12021312

[223] Cantaert T Brouard S Thurlings RM Pallier A Salinas GF Braud C. Alterations of the synovial T cell repertoire in anti-citrullinated protein antibody-positive rheumatoid arthritis. Arthritis Rheum. (2009) 60:1944–56. doi: 10.1002/art.24635 19565497

[224] Takemura S Braun A Crowson C Kurtin PJ Cofield RH O’Fallon WM. Lymphoid neogenesis in rheumatoid synovitis. J Immunol. (2001) 167:1072–80. doi: 10.4049/jimmunol.167.2.1072 11441118

[225] Klaasen R Thurlings RM Wijbrandts CA van Kuijk AW Baeten D Gerlag DM. The relationship between synovial lymphocyte aggregates and the clinical response to infliximab in rheumatoid arthritis: a prospective study. Arthritis Rheum. (2009) 60:3217–24. doi: 10.1002/art.24913 19877042

